# Exploring Unspecific Peroxygenase Selectivity with Diverse Hydrocarbon Substrates

**DOI:** 10.1002/open.202400521

**Published:** 2025-06-09

**Authors:** Essi Rytkönen, Nina Hakulinen, Janne Jänis, Juha Rouvinen

**Affiliations:** ^1^ Department of Chemistry University of Eastern Finland P.O. Box 111 FI-80101 Joensuu Finland

**Keywords:** Hydrocarbons, Hydroxylation, Oxyfunctionalization, Selectivity, Unspecific peroxygenase

## Abstract

Unspecific peroxygenases (UPOs) are enzymes capable of oxidising various substrates using hydrogen peroxide as a co‐substrate. UPOs have gained interest due to their broad substrate specificity, which includes relatively inexpensive precursors for valuable compounds, such as pharmaceuticals and chemical building blocks. In this study, the activity of a panel of 30 UPOs toward aliphatic and aromatic hydrocarbons was screened to assess variation in substrate selectivity. Most of the studied UPOs were able to oxidise the substrates efficiently, producing 2–5 products via hydrogen abstraction or epoxidation. Overall trends were observed, such as the preference for aromatic oxidation over benzylic hydroxylation in the case of toluene, while the opposite was observed with ethylbenzene. Different UPOs could also be categorized based on their reaction profiles with all substrates. Selectivity was generally low, as oxyfunctionalization reactions led to mixtures of products that were further oxidised. However, styrene and cyclohexene were converted rather exclusively to their epoxide products. The results indicate that the substrate scope of UPOs includes various types of hydrocarbons, which are oxidised depending on the individual enzyme's active site. They also hold potential for producing chemicals for industrial purposes more sustainably, but their selectivity should first be improved through protein engineering.

## Graphical abstract

Unspecific peroxygenases (UPOs) are versatile enzymes that can be used for different reactions. However, selectivity of UPOs needs to be evaluated for the desired application. Thus, the selectivity of a panel of UPOs was evaluated in this work, and it revealed diverse reactivity profiles depending on the enzyme and substrate.

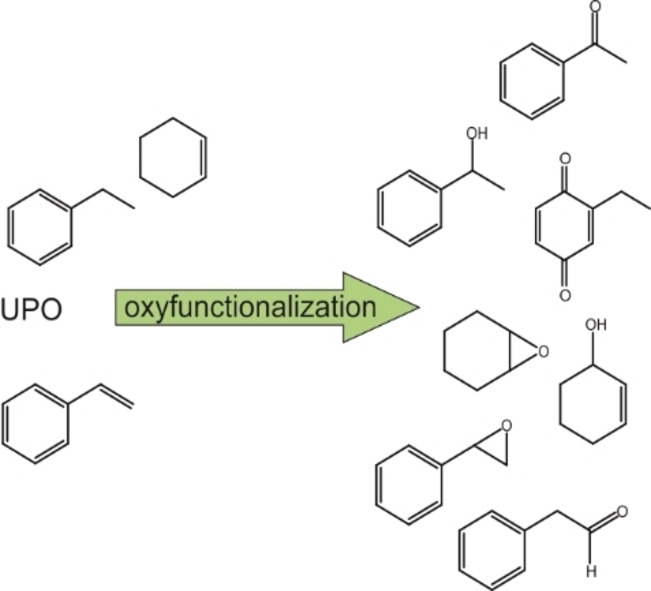

## Introduction

Unspecific peroxygenases (UPOs) are fungal heme‐thiolate enzymes capable of catalysing traditionally challenging oxyfunctionalization reactions, using hydrogen peroxide as the sole co‐substrate. The first of these enzymes was found from the fungus *Cyclocybe* (*Agrocybe*) *aegerita*, and it was originally classified as a haloperoxidase.^[1]^ However, it was reclassified as a member of a new sub‐subclass of oxidoreductases (EC 1.11.2.1), after it was recognised to oxidise alkanes among other various substrates that currently include hundreds of compounds.[^2^, ^3^, ^4^, ^5^] Over the years, more UPOs have been found from other fungi, such as *Marasmius rotula*,^[6]^ and they have been expressed as recombinants for example from *Hypoxylon* sp. EC38[^2^, ^7^] and *Coprinopsis cinerea*.^[8]^ UPOs have been found to oxidise various compounds, including alkanes,[^4^, ^9^] alkenes,^[10]^ and small aromatic compounds.^[11]^ In addition, they are quite resistant to solvents and other physicochemical factors.^[3]^ Thus, UPOs have attracted interest in many applications, such as producing industrially relevant chemicals or their precursors in a more environmentally friendly way.[^5^, ^8^] Still, there are challenges to overcome before they can reach industrial scale, for instance poor enzyme expression levels and product overoxidation.^[12]^ Applications and expression of UPOs have nevertheless advanced greatly since their discovery, and they are currently available as ready enzyme kits.[^12^, ^13^]

UPOs are a diverse group of enzymes that can be divided to two classes, long and short UPOs.[^14^, ^15^] The members of both classes share structural similarities, such as a cysteine‐bound heme, which acts as a prosthetic group, and Mg^2+^ ion, which is possibly related to the co‐factor binding.^[5]^ However, the two UPO classes differ in size (long UPOs are ∼44 kDa, while short UPOs are ∼29 kDa), but also in their substrate scope and activity due to variation in catalytic sites.[^14^, ^16^] For example, long UPOs have been observed to catalyse hydroxylation of benzene‐ring containing substrates with higher efficiency than short UPO enzymes.[^3^, ^17^] Long UPOs have substrate channels that are composed of flat aromatic residues, which confers a preference for small aromatic substrates, whereas wider active sites of short UPOs can accept bulkier substrates.^[16]^ UPOs from different species within the same class also have differences in reactivity towards the same substrate based on their active site structures.[^3^, ^18^, ^19^] For example, enantioselectivity of *Galerina marginata* UPO (*Gma*UPO) has been determined to be lower than *Aae*UPO from *C. aegerita*, although both have similar range of substrates and belong to the class of long UPOs.^[18]^

The formation of a multitude of products is one issue concerning the application of UPOs in larger scale.[^12^, ^16^] Often, UPOs produce a mixture of different oxidised compounds from single substrate, and the products can be further oxidised in subsequent reactions. For example, aromatic substrates can be oxidised on side chain, the ring or both depending on the starting compound.^[16]^ However, in industrial applications, one product is usually desired to avoid additional separation steps. Additionally, the selectivity of is dependent on the substrate, making it important to screen the wide variety of UPOs for the desired biotransformation.^[20]^ Thus, characterization of UPO selectivity will greatly aid selection of suitable enzymes for different applications.

**Figure 1:**
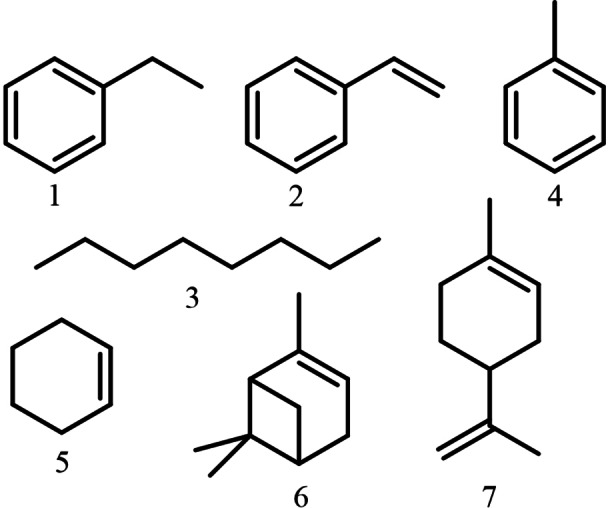
Structures of the studied hydrocarbon substrates: ethylbenzene (1), styrene (2), octane (3), toluene (4), cyclohexene (5), α‐pinene (6) and (S)‐limonene (7).

The substrate scope of UPOs is wide, thus different types of hydrocarbon substrates were selected for this study, including alkanes, small aromatics and terpenes. These included compounds that in their native or oxidised forms would be interesting as platform chemicals. The aim of this study was to characterize differences between activity of a panel of 30 UPOs towards several hydrocarbon substrates. Differences in product selectivity for each substrate between UPOs were also evaluated. Small aromatic compounds, such as ethylbenzene, toluene and styrene, were selected to study the prevalence of benzylic and aromatic oxidation. The effect of double bond in oxyfunctionalization was evaluated using styrene, cyclohexene and two terpenes, limonene and α‐pinene. In addition, octane, a linear *n*‐alkane, was tested to determine regioselectivity and reactivity in traditionally challenging oxyfunctionalization reaction. The structures of chosen substrates are presented in Figure 1.

## Results and Discussion

### Ethylbenzene

All the active UPO enzymes in the panel showed activity towards ethylbenzene, forming two to four products (Table 1). Both aromatic and benzylic oxidation products were observed. The main product of ethylbenzene oxyfunctionalization was 1‐phenylethanol, produced by all UPOs. The overoxidation product, acetophenone, was detected in lower amounts. When compared to the other panel enzymes, these two peaks for UPO16 had low intensities, indicating low conversion. In addition, UPO21 demonstrated terminal hydroxylation of the side chain (Figure S9), leading to 2‐phenylethanol and phenylacetaldehyde. UPO18 also produced small amounts of 2‐phenylethanol, but terminal hydroxylation was not observed with other panel UPOs. Two other doubly oxidised products were also observed for most of the UPOs. The first one was identified as 2‐ethyl‐1,4‐benzoquinone, which is proposed to form through two consecutive aromatic hydroxylations and subsequent oxidation to quinone (Figure S9).^[21]^ The identification of the other additional product was attempted based on the standards and published GC−MS data, but no match was found. It was concluded that the main peak in the mass spectrum (*m/z* 125.0612, corresponding to the ionic formula of C_7_H_9_O_2_^+^; Figure S3) is most likely not the molecular ion but a fragment, whose parent compound could not be determined. However, this fragment is proposed to form through ring oxidation, as it was detected only when the quinone product was present.

**Table 1:** Products observed for the UPO panel enzymes and their relative average proportions (%)^[a]^ with ethylbenzene as the substrate.

	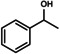	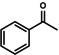	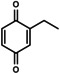	?	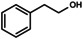	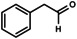
UPO	1‐phenylethanol	Acetophenone	2‐ethyl‐1,4‐benzoquinone	Unidentified	2‐phenylethanol	Phenylacetaldehyde
1	90–92	8–10	–	–	–	–
2	24–28	4	67–70	1	–	–
3	59–62	13–22	19–23	1–2	–	–
4	81–85	15–19	–	–	–	–
5	28–30	5	63–66	1–2	–	–
6	93–95	5–7	–	–	–	–
8	36–41	12–13	46–52	–	–	–
9	89–93	5–8	2	–	–	–
10	42–48	6–8	33–42	10–11	–	–
11	46–51	6–8	33–41	5–9	–	–
12	94–95	5–6	–	–	–	–
13	15–17	3	62–64	18–19	–	–
14	77	22	0.8–1.2	0.2–0.5	–	–
15	27–31	7–8	47–64	2–15	–	–
16	41–42	58–59	–	–	–	–
17	58	27	14	1	–	–
18	83–88	10–15	–	–	1–2	‐
20	31–35	4	48–55	10–13	–	–
21	20	5–7	–	–	10–18	58–64
22	84–87	9–12	1–3	1–2	–	–
23	59	40	1	–	–	–
24	50–54	11	34–38	1	–	–
13 M1	13–17	2–3	60–61	20–24	–	–
13 M2	15	2–3	63–66	17–19	–	–
13 M3	13	3–5	67–69	13–18	–	–
13 M4	15–17	3	50–64	18–29	–	–
13 M5	13–14	3–4	64–66	17–20	–	–
13 M6	13–14	3	59–68	15–24	–	–

[a] Product distributions calculated based the EIC chromatograms for the products (i. e. *m/z* 105 for 1/2‐phenylethanol, *m/z* 121 for acetophenone and phenylacetaldehyde, *m/z* 137 for 2‐ethyl‐1,4‐benzoquinone and *m/z* 125 for the unidentified product).

Ethylbenzene oxidation can occur either on the alkyl chain or aromatic ring, both options being represented in the products. Ethyl group hydroxylation led to 1‐phenylethanol and 2‐phenylethanol, which were further oxidised to acetophenone and phenylacetaldehyde, respectively. Further oxidation of aldehyde product to phenylacetic acid was not observed, however. The hydroxylation of secondary carbon atom was preferred, as only 2 UPOs demonstrated oxidation of methyl carbon. Even though benzylic oxidation was the prevalent oxidation pathway with all UPO enzymes, aromatic oxidation occurred with approximately two thirds of them. UPOs also participated in ring oxidation less prominently by forming 2‐ethyl‐1,4‐benzoquinone. The quinone is an overoxidised form of 2‐ethylbenzene‐1,4‐diol which was not observed in the reaction mixtures of panel UPOs. Conversion to quinone presumably occurs in a rapid single‐electron oxidation by peroxidase activity of UPOs,^[22]^ which would result in not detecting phenol or diol products that are intermediates in the pathway.

Ethylbenzene is possibly the most common test substrate for UPOs. For instance, *Aae*UPO hydroxylated ethylbenzene to (*R*)‐1‐phenylethanol with high enantiomeric excess (>99 %), and only traces of acetophenone were detected.^[11]^ A mutant of *Aae*UPO, PaDa−I, led to higher overoxidation with 44 % of acetophenone and the rest being 1‐phenylethanol.^[21]^ Four other UPOs have also indicated benzylic oxidation of ethylbenzene, although to lesser extent than with *Aae*UPO.^[19]^ In a study with unspecific peroxygenase from *Hypoxylon* sp. EC38 (*Hsp*UPO), three products, including 1‐phenylethanol, acetophenone and an unidentified product with a mass of 136 Da, were formed.^[2]^ It could be assumed that the unidentified product with *Hsp*UPO was 2‐ethyl‐1,4‐benzoquinone (C_8_H_8_O_2_) since similar results were obtained in this work. Aromatic hydroxylation was also detected with *Aspergillus brasiliensis* UPO (*Abr*UPO), that produced 2‐ethylphenol and 2‐ethylbenzene‐1,4‐diol in addition to quinone and benzylic products.^[21]^

Compared to these, the present results with the UPO panel and ethylbenzene as the substrate seem quite reasonable. The diverse set of products and their ratios for different UPOs can be expected due to variations in their active sites, such as the amino acid residue composition and shapes.[^21^, ^23^] The preference for benzylic or aromatic oxidation is dependent on the chain length of the alkyl substituent as well. For instance, the ring oxidation does not seem to occur with most well‐studied UPOs such as *Aae*UPO or *Mth*UPO from *Myceliophthora thermophila*.[^19^, ^21^] Aromatic products have been reported less frequently in previous studies, but here they were quite prevalent among the panel enzymes, although benzylic hydroxylation was the dominant route. In contrast to previous results, benzylic hydroxylation occurred also at a terminal carbon with the panel enzymes UPO18 and 21.

Benzylic overoxidation to acetophenone was not prominent with all UPOs, which could be due to low enzyme affinity towards 1‐phenylethanol. In fact, it has been concluded that overoxidation is dependent on the substrate and the enzyme.^[21]^ For example, *Hsp*UPO transforms 1‐phenylethanol to acetophenone almost completely, which would lead to highly efficient oxidation of the formed alcohol in ethylbenzene biotransformations.^[2]^ However, *Abr*UPO generally did not overoxidise its substrates to high extent.^[21]^ A relatively high occurrence of acetophenone in this study could also be explained by the longer reaction times. The reaction time in this study was significantly longer than for example in used in the *Aae*UPO study (10 min), which allows more overoxidation to occur.[^11^, ^23^] Additionally, the formation of acetophenone was previously influenced by the presence of acetonitrile in the reaction mixture, with higher acetonitrile content (about 30–60 vol%) resulting in less ketone product.^[24]^ Therefore, reducing the acetonitrile content could promote more prominent acetophenone formation.

### Styrene

The tested UPOs were also capable of styrene oxidation, which yielded two products with all active panel enzymes. Table 2 presents product distribution data for styrene biotransformations. The two products were identified as styrene oxide and phenylacetaldehyde, with styrene oxide being the main product. Styrene reacted only via its alkyl chain, showing no evidence of aromatic oxidation, contrary to ethylbenzene (Figure S10). All the UPOs oxidised styrene in a similar manner, differing only in product ratios, which were also quite close to each other in most cases.

**Table 2:** Products observed for the UPO panel enzymes and their relative average proportions (%)^[a]^ with styrene as the substrate.

	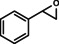	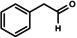
UPO	Styrene Oxide	Phenylacetaldehyde
1	58–72	28–42
2	43–51	49–57
3	62–72	28–38
4	70–73	27–30
5	39–51	49–61
6	47–52	48–53
8	37–50	50–63
9	69	31
10	42–60	40–58
11	44–52	48–56
12	66–75	25–34
13	52–59	41–48
14	64–71	29–36
15	34–46	54–66
16	66–76	24–34
17	47–57	43–53
18	64–77	23–36
20	32–50	50–68
21	67–70	30–33
22	60–72	28–40
23	63–73	27–37
24	52–62	38–48
13 M1	56–62	38–44
13 M2	44–56	44–56
13 M3	49–64	36–51
13 M4	47–66	34–53
13 M5	47–60	40–53
13 M6	49–61	39–51

[a] Based on the FID data.

Product ratios differed slightly between the enzymes, with styrene oxide amount being 32–77 % of total products in individual experiments. Phenylacetaldehyde was observed in all reaction mixtures, but its presence could be due to isomerisation of styrene oxide through Meinwald rearrangement instead of enzymatic conversion.[^21^, ^25^] Some epoxides are not stable, and spontaneously isomerise to their carbonyl counterparts via ring opening.^[26]^ Styrene oxide has been observed to rearrange to phenylacetaldehyde, especially in elevated temperatures, and can occur for example in GC‐liners.^[27]^ Therefore, the presence of phenylacetaldehyde may be partly attributed to the analysis conditions. However, the aldehyde yields for the UPOs varied and were quite high in some cases, differing considerably from the epoxide‐to‐aldehyde ratio of the styrene oxide standard measured in the same conditions. It has recently been determined that UPOs can convert styrene derivatives to aldehydes through anti‐Markovnikov Wacker‐type oxidation.^[28]^ In the work of Swoboda et al., it was concluded that the aldehyde products do not form through Meinwald arrangement from the epoxide, but through a separate minor reaction route.^[28]^ Thus, it is likely that the UPO panel enzymes can also produce phenylacetaldehyde directly from styrene, since it was even observed as the major product for some of them.

The styrene reactivity has previously been studied with several UPOs, for example *Aae*UPO[^11^, ^29^] and *Hsp*UPO,^[2]^ using various reaction set‐ups. As in this study, only side chain oxidation has been observed. Using *Hsp*UPO, styrene was almost completely converted to styrene oxide and phenylacetaldehyde with product ratios of 91 % and 9 %, respectively, whereas for *Aae*UPO, conversion rate to epoxide was 71 %.[^2^, ^11^] Both enzymes produced only low enantiomeric excess of the epoxide, which has been hypothesised to be due to lack of hydrogen bonding to protein groups in the binding mode.^[2]^ Immobilized *Aae*UPO PaDa−I mutant in neat conditions has also been an efficient oxidation catalyst, converting styrene to epoxide (88 %) and aldehyde (12 %) through rearrangement, similar to *Abr*UPO that showed minor isomerisation of the epoxide product.[^21^, ^26^] In general, *Aae*UPO has a high styrene activity, as it exhibited the highest turnover number (TON) of five enzymes.^[19]^ However, *Chaetomium globosum* UPO (*Cgl*UPO) had almost comparable TON and much higher enantioselectivity of 44 %, whereas *Mth*UPO had the highest enantioselectivity with 45 % of (*S*)‐epoxide but notably smaller TON.^[19]^ Additionally, mutations in the active site have been proven to affect the turnover number and stereoselectivity of the *Mth*UPO‐catalysed styrene epoxidation.^[27]^ Enantioselectivity was not determined in this study, but similar preference for epoxidation was found, although the higher aldehyde proportions could form via a separate reaction route, as proposed recently.^[28]^

### Octane

Octane oxyfunctionalization has been deemed challenging due to inert C−H bonds, but several products were observed in this work with varying oxidation levels and positions, depending on the used enzyme. Overall, the products included C2‐, C3‐ and C4‐oxidised compounds (i. e., alcohols and ketones), but terminal oxidation was not detected. The found products for octane biotransformations are presented in Table 3. The number of products per enzyme varied from one to five, with oxidation occurring in 1–3 different carbon atoms.

**Table 3:** Products observed for the UPO panel enzymes and their relative average proportions (%) with octane as the substrate.

			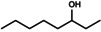		
	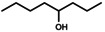	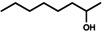	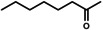	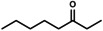	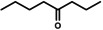
UPO	2‐octanol	3‐octanol	4‐octanol or 2‐octanone^[a]^	3‐octanone	4‐octanone
1^[b]^	30–45	30–34	21–39	–	–
2	–	n. d.	n. d.	n. d.	n. d.
3^[b]^	16–100	0–21	0–46	0–17	–
4^[b]^	22–49	26	26–52	–	–
5^[b]^	–	–	20–21	32–44	36–47
6^[c]^	100	–	–	–	–
8^[b]^	–	15–22	34–35	21–31	20–22
9^[c]^	39–46	30	24–32	–	–
10^[b]^	–	–	23–27	37–43	35–37
11^[c]^	20–31	22–26	27–32	3–4	17
12^[b]^	63–70	30–37	–	–	–
13	n.d.	–	n. d.	n. d.	n. d.
14^[b]^	–	0–11	0–11	27–70	30–52
15	n.d.	n.d.	n.d.	n.d.	n.d.
16	–	–	–	–	–
17^[b]^	–	3–6	9–17	14–15	63–73
18^[b]^	–	0–10	78–100	0–12	–
20	–	n.d.	n.d.	n.d.	n.d.
21	–	–	–	–	–
22^[b]^	6–10	10–19	23–36	13–23	21–34
23^[b]^	–	22–30	26–31	44–47	‐
24^[b]^	–	0–4	43–46	28–31	22–26
13 M1^[b]^	–	0–7	23–26	16–18	49–61
13 M2^[b]^	–	0–6	18–26	18–20	56
13 M3^[b]^	–	0–6	19–23	21	54–56
13 M4^[b]^	–	0–7	16–36	14–30	34–63
13 M5^[b]^	–	0–5	18–21	21–24	55–57
13 M6^[b]^	–	0–7	19–24	12–21	57–59

[a] 4‐Octanol and 2‐octanone are listed as one product since their retention times were similar and they could not be distinguished by MS fragments in the reaction spectra, [b] Relative amounts (%) calculated from EIC *m/z* 107 chromatograms, [c] Relative amounts (%) have been calculated from FID chromatogram areas, n. d.=not determined.

Both octanols and octanones were observed in the reaction mixtures, and oxidation of product alcohols to corresponding ketones occurred in most cases. A trend observed for oxidation was towards the secondary carbons, *i*. *e*. C4‐ and C3‐oxidation products were the most abundant whereas C2‐oxidation appeared to occur less frequently. The terminal oxidation of octane (to 1‐octanol or octanal) was not observed for any of the UPO enzymes. In general, the product quantities were small based on the ion abundances, possibly owing to the low substrate solubility and/or decreasing oxyfunctionalization efficiency with an increasing chain length of alkane.^[9]^

Based on the previous studies, the oxyfunctionalization of alkanes with UPOs often occurs in the secondary C2‐ and C3‐positions, and terminal oxidation has rarely been reported.[^9^, ^27^] For instance, octane oxyfunctionalization with *Mth*UPO resulted in 2‐, 3‐ and 4‐octanols, out of which reaction with C3 was the most prominent one, followed by C2.^[27]^ The regioselectivity of the enzyme could be altered with mutations, either towards 4‐octanol or terminal hydroxylation, which has otherwise been observed only with *Mro*UPO from *M. rotula*.[^27^, ^30^] For *Aae*UPO, the transformation products of octane were 2‐ and 3‐octanols, in addition to about 1 % of ketones.^[9]^ The same alcohol products were also observed for UPO from *Thielavia terrestris*, whereas *Gma*UPO demonstrated no activity.^[27]^ In another study, *Gma*UPO and *C. cinerea* UPO (*Cci*UPO) variants transformed octane to 2‐ and 3‐octanols with corresponding ketones.^[31]^ *Aae*UPO has also produced dihydroxylated products at C2 and C3, as well as combinations thereof, including both hydroxyl and carbonyl groups in *n*‐alkanes with 12–16 carbon atoms.^[4]^ *Cci*UPO gave comparable results with tetradecane.^[8]^ Consequently, regioselectivity does not seem to be dependent on the alkane chain length, but it slightly varies between UPOs.[^4^, ^5^, ^9^] Nonetheless, it has been said that *Aae*UPO converts octane quite poorly,^[32]^ which was true for UPO panel enzymes as well.

The previous results of alkane oxidation with UPOs agree with the regioselectivity of the obtained products. Based on the product trends, UPOs seem to favour the internal carbons over terminal ones. This has led mostly to C2‐ and C3‐hydroxylated products, although the C4‐position seemed to be the prominent one for octane in this study. However, 4‐octanol and 2‐octanone were indistinguishable based on their retention times and fragmentation patterns in APCI, which could lead to false interpretation of the oxidation position. Both products could also be present, depending on the UPO enzyme. The rarity of terminal oxidation is understandable based on the high bond strengths in alkanes.^[30]^ Also, the proposed binding mode of octane makes positions C2, C3 and C4 available for oxidation.^[27]^ Regardless, all the products were present mostly only in trace levels and several oxidation positions were identified for each UPO, resulting in low selectivity.

The earlier studies on alkane oxyfunctionalization have mostly reported only alcohol products but no further oxidation to carbonyl compounds. However, with the protocol used for octane, overoxidation to ketones was often detected, which could be due to the reaction time in addition to the enzyme's ability to further oxidise alcohol products. The 4 h reaction time probably allowed the formation of carbonyl products, whereas these products were not observed with shorter reaction times used in the previous studies, for example, 1 h used by Knorrscheidt et al.^[27]^ and Babot et al.^[8]^ The high occurrence of ketones could be also explained by the substrate availability. Octane is only slightly water‐soluble and not as accessible to enzyme in the aqueous phase. Alcohol products are more soluble and are thus preferred substrates as compared to alkanes.^[33]^ Octanols also remain in the active site (following their formation) since they are not replaced by octane very rapidly, and are likely converted further.^[33]^ As with ethylbenzene, a low acetonitrile content in the reaction mixture could also promote the formation of ketones.^[24]^

### Toluene

Toluene is a small aromatic molecule with only one carbon in the side chain. However, its reactivity differed significantly from that of ethylbenzene (which has two carbon atoms in the substituent), with major oxidation occurring at the ring carbons for most UPO enzymes. This likely results from the C−H bond energies, being slightly lower for the benzylic carbon in ethylbenzene (83 kcal/mol) as compared to the primary carbon in toluene (87 kcal/mol) based on quantum chemical calculations.^[22]^ Benzylic oxidation occurred to the lesser extent and was not observed for all the UPOs. The products for toluene biotransformations are listed in Table 4. The reaction routes for toluene oxyfunctionalization are proposed in Figure S11.

**Table 4:** Products observed for the UPO panel enzymes and their relative average proportions (%)^[a]^ with toluene as the substrate.

	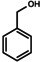	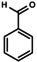	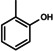	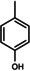	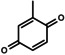
UPO	Benzyl Alcohol^[b]^	Benzaldehyde^[b]^	*o*‐cresol^[b]^	*p*‐cresol^[b]^	Methyl‐*p*‐benzoquinone^[c]^
1	–	–	82–100	0–18	–
2	–	–	20–40	0–4	60–77
3	–	–	23–52	10–17	31–67
4	–	–	88–100	0–12	–
5	–	–	15–39	0–5	56–85
6	–	–	53–55	45–47	–
8	–	–	81–100	–	0–19
9	0–2	–	77–80	15–20	0–7
10	–	–	49–72	8–10	20–42
11	–	–	42–67	–	33–58
12	0–2	0–2	82–87	3–5	5–15
13	0–0.4	2	2	1	95
14	23–33	23–67	0–9	0–7	0–37
15	–	–	71–72	5	23–25
16	–	–	–	–	–
17	0–6	0–8	29–39	9–16	45–48
18	11–13	87–89	–	–	–
20	–	–	51–61	0–5	34–49
21	3–8	92–97	–	–	–
22	–	–	–	–	–
23	0.4–2	6–7	0.1–0.6	0.1–0.3	91–92
24	1–2	3	12–19	2–4	73–82
13 M1	–	0–2	5–6	1–36	58–92
13 M2	–	1–2	6–15	2	81–91
13 M3	–	2–3	0.6–1	0–3	95–96
13 M4	–	1–2	6–25	2–5	69–90
13 M5	–	2–3	1–4	0–2	92–96
13 M6	–	1–2	4–18	1–3	78–93

[a] Calculated from the EICs for the products. [b] Identified based on the authentic standard. [c] Identified by accurate mass (elemental formula).

Toluene was converted to mixture of *o*‐ and *p*‐cresols in aromatic oxidation, which presumably proceed through epoxidized intermediates. The *ortho*‐position was the favoured one with mostly minor amounts of *para*‐hydroxylation products detected. Methyl‐*p*‐benzoquinone was also observed with most UPOs, forming via a transient methyl hydroquinone intermediate that results from aromatic hydroxylation of *o*‐cresol. Methyl hydroquinone (a diol) is not stable and was not observed in the experiments. Furthermore, benzylic hydroxylation of toluene led to benzyl alcohol which was further oxidised to benzaldehyde. The presence of overoxidation products (mostly benzaldehyde) in the absence of alcohol indicates fast second oxidation and preferable conditions for it.

Previously, toluene has been oxidised to benzyl alcohol, benzaldehyde, benzoic acid, *o*‐cresol, *p*‐cresol and methyl‐*p*‐benzoquinone by *Aae*UPO.[^34^, ^35^] The lack of selectivity likely results from different binding modes of toluene to the active site, which allows oxidation of both side chain and aromatic ring carbons.^[36]^ Nevertheless, *Aae*UPO preferred alkyl oxidation and aromatic oxidation contributed approximately one third of the products.^[37]^ Similarly, oxidation with *Mro*UPO occurred mostly in the benzylic positions with the main products being benzoic acid (89 %) and benzaldehyde (7.3 %). A minor amount of methyl‐*p*‐benzoquinone (3.6 %) was also produced.^[6]^ Using toluene derivatives as a substrate, the selectivity of *Aae*UPO could be shifted exclusively to benzylic oxidation.^[36]^ Contrary to *Aae*UPO and *Mro*UPO, aromatic oxidation seemed to be the major pathway for toluene oxidation among all the UPOs in this study. Only three UPOs preferred the benzylic route, and aromatic oxidation was not observed at all for two of those. Furthermore, benzoic acid was absent in analyses, although its presence was be expected based on previous studies. Otherwise, similar products to those reported in earlier studies were obtained.

### Cyclohexene

Cyclohexene is a cyclic hydrocarbon with a double bond that can undergo epoxidation or allylic hydroxylation to form alcohols (Figure S12). Cyclohexene was converted rather selectively to the epoxide product, cyclohexene oxide, by all UPOs. A few enzymes also demonstrated ability to form allylic alcohol 2‐cyclohexen‐1‐ol and the corresponding ketone, 2‐cyclohexen‐1‐one, by further oxidation. The observed products and their relative amounts are presented in Table 5. The alcohol product was observed only in the second screen for some UPOs, which could be due to difficulty in detection of small product amounts. For UPO18, this cannot be the case as alcohol was present in substantial amount, and it was fully overoxidised to ketone in the first screen. Thus, overoxidation has not been as efficient, possibly due to lack of peroxide which could result from reaction mixture heterogeneity, as the conditions were same in both screens.

**Table 5:** Products observed for the UPO panel enzymes and their relative average proportions (%)^[a]^ with cyclohexene as the substrate.

	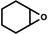	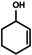	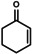
UPO	Cyclohexene Oxide^[b]^	2‐cyclohexen‐1‐ol^[c]^	2‐cyclohexen‐1‐one^[c]^
1	100	–	–
2	100	–	–
3	100	–	–
4	100	–	–
5	100	–	–
6	92	8.1	–
8	100	–	–
9	96	3.9	–
10	98–100	0–2.0	–
11	100	–	–
12	63	37	–
13	99–100	0–1.4	–
14	97	3.1	–
15	99–100	0–1.5	–
16	93	6.5	–
17	96–97	0–1.4	2.7
18	65–68	0–16	19–32
20	97–100	0–3.3	–
21	73	5.3	22
22	100	–	–
23	100	–	–
24	98	–	2.2
13 M1	99–100	0–1.4	–
13 M2	99	1.4	–
13 M3	99–100	0–1.2	–
13 M4	99–100	0–1.1	–
13 M5	100	–	–
13 M6	100	–	–

[a] Based on the FID chromatograms. [b] Identified based on authentic standard. [c] Identified based on linear retention index/mass spectra.

In the previous studies, epoxidation was the preferred oxidation route for cyclohexene together with a minor allylic hydroxylation.^[37]^ The formation of the epoxide product has been suggested to be energetically more favourable based on quantum mechanical calculations.^[27]^ For example, biotransformations with *Aae*UPO yielded cyclohexene oxide (55 %) and 2‐cyclohexen‐1‐ol (45 %) as the products.^[10]^ Photocatalytic reactions with *Aae*UPO also converted cyclohexene to cyclohexene oxide and 2‐cyclohexen‐1‐ol with rather high TONs.^[32]^ For *Agaricus bisporus* var. *bisporus* UPO and *Gma*UPO, only the epoxidation product was observed, whereas *Mth*UPO variants additionally produced low amounts of allylic alcohol.[^18^, ^27^, ^38^] Single‐point mutations were insufficient to shift *Mth*UPO selectivity toward allylic hydroxylation, suggesting that additional mutations are required to alter the reaction pathway.^[27]^ In addition to epoxide, *Hsp*UPO oxidation products included epoxy ketone and benzoquinone, which were proposed to form through allylic hydroxylation.^[2]^ The results from UPO panel experiments align with these findings, as cyclohexene oxide was the major product, and for twelve of the UPOs it was also a sole product. The alcohol product was observed with roughly half of the UPOs, and a third product, a ketone, was additionally detected in minor amounts The overoxidation was uncommon, except with the UPO panel enzymes 18 and 21.

### α‐Pinene

*α*‐Pinene is a bicyclic monoterpene abundantly found in nature, for instance, in plant essential oils and the resins of coniferous trees.^[39]^ It is also an important chemical for the production of other chemicals used in cosmetics and food products, such as verbenol and campholenic aldehyde.[^40^, ^41^] α‐Pinene is a major compound found in turpentine, a by‐product of chemical pulping, which makes its biotransformation into value‐added compounds economically beneficial.[^40^, ^42^] The oxidation of α‐pinene proceeds via two routes, allylic hydroxylation or double bond epoxidation (Figure S13).^[40]^ Both reaction types were utilised by the UPO panel enzymes, as shown by the products in Table S1.

The main oxidation pathway in the reactions was allylic hydroxylation with verbenol and verbenone as the major detected products. Other allylic products were also identified. Epoxidation converted α‐pinene to α‐pinene oxide, which was rearranged to camphenol and campholenic aldehyde. However, possible oxidation products, excluding verbenol and α‐pinene oxide, were also present as artefacts in negative control reactions and the pinene standard. Autoxidation of α‐pinene to terpenoids, such as verbenone and myrtenal, has been observed previously.^[43]^ As these products were observed in the experiments without enzyme and peroxide, they were considered as enzymatic oxidation products only if their peak area was more than twice the area in the negative controls. The arbitrary limit was set to clearly distinguish autoxidation of the substrate from enzymatic activity, and it may have caused differences between detected products between the two screens.

Previously, *Abr*UPO has been reported to efficiently oxidise α‐pinene, forming a product mixture whose composition was not reported.^[21]^ In the biphasic system, *Aae*UPO mutant PaDa−I was able to convert α‐pinene to various terpenoids through epoxidation and hydroxylation.^[40]^ The main product which favoured allylic hydroxylation was verbenol, but epoxidation led to various side reactions over longer reaction times to produce other compounds from α‐pinene oxide.^[40]^ Additionally, both PaDa−I and artificial artUPO produced *trans*‐sobrerol as the main product through α‐pinene oxide, with rearrangement of epoxide to campholenic aldehyde, and the side reaction to verbenone by artUPO.^[44]^ Here, oxyfunctionalization also led to complex mixture of compounds with varying levels of oxidation. Verbenol and campholenic aldehyde were frequently detected, similar to previous studies. However, identification solely based on retention indices was challenging since there were several compounds that were in the acceptable LRI range (Table S2). Additionally, the obtained mass spectra were rather similar and not informative unless compared to the authentic standards (e. g. myrtenal and α‐terpineol). Nevertheless, UPO biotransformations did not lead to neither efficient nor selective oxidation of α‐pinene and protein engineering would be needed to finetune the selectivity towards the desired compounds.

### (*S*)‐Limonene

Limonene enantiomers, (*R*)‐limonene and (*S*)‐limonene, are naturally abundant monoterpenes for which the oxidised derivatives are industrially interesting compounds, e. g. in food products.[^10^, ^45^] The (*S*)‐enantiomer (used in this study) is found for example in *Pinus* trees and some herbs.[^42^, ^45^] A selective biotransformation of limonene is desirable since the cheap raw material could be easily converted to valuable compounds, such as anti‐tumour agent perillyl alcohol.[^45^, ^46^] As for α‐pinene, there seems to be two distinct reaction mechanisms for limonene (Figure S14), allylic oxidation to form alcohols and carbonyls and epoxidation of the double bond.

(*S*)‐limonene was converted to the mixture of products that differed between UPOs (Table S4). Five products were identified, including 8,9‐epoxylimonene, carveol and perillyl alcohol. Perillyl alcohol was present with most UPOs, and it was overoxidised to aldehyde by a few enzymes. In addition, limonene dioxide was detected only for UPO21. Another possible product peak detected with few UPOs could tentatively be identified as limonene‐1,2‐diol, based on LRI data (Table S3). However, the mass spectrum of the compound did not fully correspond to the diol spectra in the database, so it was not included in identification tables. Furthermore, identification based on LRIs was challenging, especially for the compounds eluting at very similar retention times (and, thus similar LRIs). For example, one of the oxidised limonene isomers observed could be identified as 8,9‐epoxylimonene or isopiperitenol, in which case the overoxidation product could also be isopiperitenone (Table S3). Nevertheless, the compound is more likely an epoxide, as the mass spectrum was identical to that of 8,9‐epoxylimonene.

Some of the proposed oxidation products, mainly epoxides, were also present in negative control reactions, which suggests that they were impurities in the substrate itself. The autoxidation of limonene in air has been observed in earlier studies,[^47^, ^48^, ^49^] with major compounds being 1,2‐epoxylimonene, carveol and carvone.^[48]^ These oxidation products were detected in the negative controls as well, indicating that autoxidation of limonene had occurred before the experiments. Thus, epoxylimonenes and carveol were only counted as the true oxidation products if their peak areas in chromatograms were twice the peak areas in the negative controls. For example, 1,2‐epoxylimonene was observed in all the reactions, including a control without enzyme and hydrogen peroxide, but it was not counted as a reaction product as it did not exceed the set boundary conditions.

The main products reported for (*S*)‐limonene were 1,2‐epoxylimonene, 8,9‐epoxylimonene and carveol in the *Aae*UPO biotransformations.^[10]^ The epoxidation was a major route for oxyfunctionalization, since 85 % of the products were epoxides.^[10]^ Moreover, photocatalytic reaction of (*R*)‐limonene with the same UPO yielded a mixture of epoxidation and allylic hydroxylation products that were possibly hydrolysed to side products.^[32]^ PaDa−I and artUPO have also been reported to produce mixture of epoxides from (*S*)‐limonene.^[44]^ However, when using *Aae*UPO PaDa−I mutant to convert limonene derived from orange peels, only allylic hydroxylation to carveol and the consequent overoxidation to carvone were observed.^[50]^ Here, both allylic alcohols and epoxides were observed, with perillyl alcohol being most widely present among different UPOs. A further oxidation to carbonyl compounds did not occur frequently or they were below detection limit, although overoxidation was observed in few experiments also for epoxide product. The epoxidation products were included in enzymatic products less frequently (due to set boundaries) even though they were frequently detected.

The products of the two screens differed slightly for most of the panel for both (*S*)‐limonene and α‐pinene, which could result from multiple reasons. First, the detection criteria for compounds in negative control causes errors in identification, as the value is only arbitrarily chosen to separate enzymatic activity from autoxidation. The detection limit may hinder product identification, and other analysis conditions may have caused challenges in detection of small product amounts, for example extraction may not have been complete. However, limonene oxidation was not highly selective, and the results indicate diverse reactions with terpenes.

### Substrate Oxidation Trends

The panel UPOs exhibited different selectivities depending on the enzyme, but certain product trends for substrate types could be observed. The substrates containing double bonds were preferentially converted to their oxidised counterparts rather efficiently. With these substrates, conversion products were formed either through epoxidation of the double bond or by allylic hydroxylation depending on the enzyme. Compared to other double bond‐containing substrates, terpene oxidations were not as prominent and resulted in complex mixtures. The double bond in aromatic system (styrene) led only to side chain oxidations, whereas with saturated alkyl substituent, aromatic oxidation was also observed and even more prominent in the case of toluene. The completely saturated substrate, octane, was converted rather poorly, but it exhibited a variety of products with oxidation trend to oxidise the “inner” carbons. The overoxidation of alcohol products to corresponding carbonyl compounds was prevalent for all substrates, except limonene, which could also result from a rather long reaction time.

Overall, selectivity of UPOs was low, although styrene and cyclohexene were rather exclusively converted to epoxide products. Especially with cyclohexene, most UPOs were selective towards cyclohexene oxide with only minor allylic oxidation. Similarly, styrene oxide was the oxidation product with all enzymes, but aldehyde also formed in rather high amounts with some UPOs. These selectivities could be utilised in larger‐scale applications of UPOs, since cyclohexene oxide and styrene oxide are valuable intermediates industrially.^[41]^ For terpenes, the selectivity was among the lowest, as one to five oxidation products could be observed for a single enzyme. The identifications of some products were also tentative because of large scope of possible oxidation products which had very similar retention indices and mass spectra. The selectivity in octane products was also low, as oxidation primarily occurred at two or three different carbon atoms, also resulting in ketone formation. Toluene was quite selectively transformed into *o*‐cresol when aromatic oxidation was the major reaction route, but it was mostly overoxidized efficiently and other products were present in lower amounts. Finally, ethylbenzene favoured formation of secondary benzylic alcohol, but aromatic oxidation was notable for two thirds of the UPOs, lowering the selectivity.

### Reactivity Profiles Between the UPO Panel Enzymes

**Figure 2:**
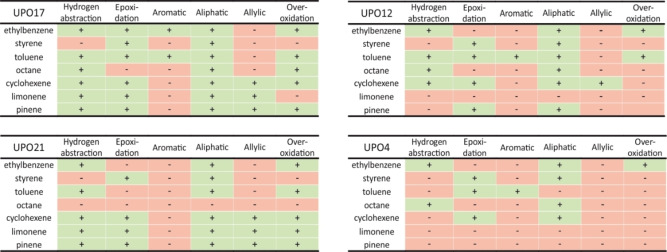
Reactivity profile fingerprints of the selected UPO panel enzymes, showcasing enzymes with diverse reactivity as well as those with more limited oxyfunctionalization potential. Fingerprints for all other UPO panel enzymes are provided in the Supplementary Information.

The UPO panel enzymes exhibited noticeably different selectivity profiles when compared to one another (Figures 2 and S2). There were three UPOs that did not show aromatic oxidation (UPOs 16, 18 and 21). Aromatic oxidation was not observed with styrene; however, the remaining UPOs oxidized the ring carbons of one or both of the other aromatic substrates. However, UPOs 1 and 4 did not seem to prefer aromatic oxidation or epoxidation, in general, when it was possible based on the substrate. Oxidation to aliphatic carbons was prevalent for all the substrates, except for toluene, for which only aromatic oxidation was observed with some enzymes. Allylic hydrogen abstraction occurred for all the substrates having allylic positions, with about half of the panel UPOs. However, UPOs 1 and 4 did not seem to utilise allylic oxidation route at all and were also inactive toward terpenes. UPOs 1, 4, 6, 12 and 22 did not extensively overoxidize the substrates, as overoxidation was only observed with ethylbenzene or with both ethylbenzene and toluene. UPO16 had low or no activity with substrates that did not contain double bonds (i. e. ethylbenzene, octane, toluene). UPO6 was highly specific to form 2‐octanol from octane, whereas other UPOs oxidised at least two separate carbon atoms. Additionally, UPO21 seemed to have a unique selectivity profile, not observed with other enzymes, for example a terminal hydroxylation of the ethylbenzene side chain.

The UPO panel enzymes could be divided to versatile and more limited ones based on their selectivity profiles. Certain UPOs exhibited low or no conversion towards several substrates, e. g., UPO22 with toluene and α‐pinene. UPOs 7 and 19 were deemed inactive, as no products were not formed for any of the studied substrates, or they were below the detection limit in all cases. All variants had similar profiles to their parent enzyme UPO13, although variants M5 and M6 did not form epoxides from limonene, which can at least partially be explained by the boundary conditions for product identification. Additionally, UPOs 17 and 24 had similar profiles to variant UPOs. These UPOs seemingly were the most active ones, reacting to several different positions and utilising both reaction routes for most of the substrates. On the other end of the activity spectrum were UPOs 1 and 4, which activity towards different routes and positions was more limited. Reactivity to less positions and utilisation of certain routes would be more beneficial for selective transformations if activity could be increased by protein engineering. Additionally, no tendency for overoxidation would imply less work in preventing further reactions of wanted products.

Differences in reactivity of UPO classes have been recognised, which may explain some similarities and contrasts between panel UPOs. In general, long UPOs preferentially oxidise small aromatic substrates, such as styrene and ethylbenzene, due to their inflexibly formed substrate channels.^[16]^ Thus, they would be the most efficient enzymes in this study, whereas short UPOs would display lower activity. Long UPOs additionally might prefer benzene ring oxidation to side‐chain oxidation for some substrates such as toluene.^[3]^ However, this has not been observed for alkylbenzenes, as for example, long *Aae*UPO produces exclusively alkyl hydroxylation products from ethylbenzene, and aromatic oxidation is only indicated with short UPOs *Hsp*UPO and *Abr*UPO that are said to barely react to aromatic rings.[^2^, ^3^, ^21^] Based on their wider and more flexible substrate channels, short UPOs would be more active with large, aliphatic substrates,[^5^, ^16^] which in this study could relate to octane activity. Nevertheless, substrate scope and activity are dependent on individual UPO's active site structure, as two UPOs from different species within the same class can have diverse results,[^3^, ^18^, ^19^] which the diversity observed in this study would also indicate.

## Conclusions

In this study, a panel of unspecific peroxygenases was screened with seven hydrocarbon substrates to study the variation in substrate scope and selectivity. The UPOs were capable of various types of oxyfunctionalization reactions, resulting in product mixtures for most of the substrates. Majority of the UPOs oxidised all seven substrates, although there were some exceptions which can be expected from a large pool of enzymes. Overall selectivity of the panel enzymes was rather low, but styrene and cyclohexene exhibited high selectivity towards epoxidation products.

Trends for preferred oxidation type could be drawn for substrates and different UPOs. Ring oxidation occurred with aromatic substrates, except for styrene, which benzylic double bond shifted selectivity towards its epoxidation. For other double bond‐containing substrates, epoxidation was also observed in addition to allylic hydroxylation. Saturated hydrocarbon chains went through hydrogen abstraction and hydroxylation to form alcohols. As for overall UPO trends, three UPOs did not participate in aromatic oxidation, whereas for others it was even preferred over aliphatic oxidation with toluene as the substrate. Nevertheless, aliphatic oxyfunctionalization was prominent with all panel UPOs. Allylic oxidation was often present with substrates including these positions, especially with terpenes. Only a few UPOs did not tend to overoxidise their products, but prevalent overoxidation could also be due to reaction conditions.

Selectivity towards a certain product would be industrially interesting, as platform chemicals from cheaper materials could be obtained more easily and environmentally friendly way. High activity on studied model substrates could relate to potential on more complex substrates, for example styrene active UPOs could possibly participate in polystyrene degradation. However, there are still many challenges to overcome before utilisation of UPOs in larger scale, such as low selectivity, but more active and selective UPOs could be derived from protein engineering.

## Experimental Section

### Materials

The panel of unspecific peroxygenases used in this study was obtained from Aminoverse (Nuth, the Netherlands). It comprised 24 recombinantly produced UPO enzymes along with 6 variants of one of the panel enzymes. The substrates were obtained from Sigma Aldrich (styrene, octane, cyclohexene, α‐(+)‐pinene and (*S*)‐(−)‐limonene), Thermo Fischer (ethylbenzene) and VWR (toluene). The reaction products were identified, if possible, using commercial standards obtained from TCI Europe (styrene oxide, 1‐phenylethanol, acetophenone, 3‐octanol and 4‐octanone), Thermo Fischer (phenylacetaldehyde), Merck (1‐octanol), LGC standards (3‐octanone), TRC Canada (2‐ethyl‐1,4‐benzoquinone) and Sigma Aldrich (*o*/*p*‐cresol, benzyl alcohol, benzaldehyde, 2‐phenylethanol, 2‐octanol, 4‐octanol, octanal, 2‐octanone, cyclohexene oxide, verbenol, verbenone, myrtenal, α‐terpineol and carveol). Cyclo‐octanol obtained from TCI Europe was used as an internal standard. The solvents (hexane and acetonitrile) were obtained from Merck and VWR Chemicals, respectively. Ammonium acetate solution was prepared from the analytical grade (≥99.99 %) reagent obtained from Honeywell. Additionally, co‐substrate solutions for the oxyfunctionalization were prepared from aqueous hydrogen peroxide solution (30 wt %) obtained from Merck.

### Oxyfunctionalization Reactions with UPOs

Reactions with UPO enzymes were carried out in 300 μL scale in 2 mL reaction tubes. The reaction mixtures contained 30 μL of the enzyme solution, 100 mM ammonium acetate (pH 7.5), and 4 mM of substrate dissolved in acetonitrile (4 μL of 200 mM stock solution). The final reaction mixture contained approximately 2 % (v/v) of acetonitrile. Hydrogen peroxide (200 mM solution, 4 μL) was added to the mixture to start the reaction, and it was added in the same volume second time at 120 minutes to reach a final concentration of 7.8 mM. Reaction mixtures were incubated in a benchtop shaker incubator (Thermomixer comfort, Eppendorf, Hamburg, Germany) at 30 °C, 500 rpm for 4 hours. Reaction mixtures were extracted in the incubator with 300 μL of *n*‐hexane, containing internal standard (cyclo‐octanol, 1.98 mM), for 30 minutes. All reactions were performed in duplicates (i. e., screens 1 and 2). Negative control reactions were performed to ascertain that the substrate was not oxidised solely by hydrogen peroxide present in the reaction mixtures. These control reactions were conducted in the same manner as the UPO reactions, except that the enzyme solution was replaced with same volume of ammonium acetate solution.

### GC−MS/FID Analysis

The hexane extracts obtained from the reaction mixtures, after being diluted 2 : 3 (v/v) with *n*‐hexane, were analysed by GC−MS/FID. All experiments were carried out on a Bruker/Scion 456‐GC gas chromatograph equipped with a PAL‐RSI autosampler, connected to a timsTOF quadrupole time‐of‐flight (Q‐TOF) mass spectrometer (Bruker Daltonics GmbH, Bremen, Germany) featuring an atmospheric pressure chemical ionization (APCI) source. The instrument was additionally equipped with a standard flame ionization detector (FID) operated at 300 °C, featuring a post‐column split to direct the output between the MS and FID detectors. The analytes were separated on a Restek RXi‐5Sil MS capillary column (30 m×0.25 mm i. d., 0.25 μm film thickness), with an injector temperature of 280 °C. A split injection was used at a split ratio of 10 : 1 and an injection volume of 1 μL. Ultrapure helium was used as a carrier gas at flow rate of 1.0 mL/min. The temperature program was as follows: 40 °C for 2 min, then 10 °C/min to 180 °C, followed by a 2 min hold time. The transfer line to the ion source was kept at 285 °C during the measurements. The APCI source was operated in the positive ion mode. The other instrument parameters were as follows: capillary voltage, 3 kV; corona needle, 3 μA; source temperature, 200 °C; nitrogen gas flow, 2.5 L/min; gas temperature 220 °C; *m/z* range, 50–800. Bruker Compass HyStar 6.0 and ot of Control 6.2 software were used for instrument control and data acquisition, and Bruker Compass DataAnalysis (version 5.1) for data post‐processing and analysis.

### Product Identifications

The reaction products were identified by comparison with authentic analytical standards or previously published data. In the former case, the standards were analyzed using GC−MS/FID under the same conditions as those used for the reaction product analysis. In the latter case, the identifications were based on the linear retention indices (LRIs), averaged from the PubChem^[51]^ database in February 2024 (“semi‐standard nonpolar column”) and by comparison with the reference mass spectra, available in NIST WebBook.^[52]^ The obtained accurate masses for the precursor and fragment ions were also utilised in the product identifications. The LRIs^[53]^ were obtained by comparing the retention times to an *n*‐alkane standard (C_8_‐C_20_, Sigma Aldrich), measured separately in identical conditions. Relative product percentages were calculated based on the chromatographic areas, obtained by FID (styrene and cyclohexene). When it was not possible to calculate relative product ratios from the FID data, extracted ion chromatograms (EICs) for selected precursor ions were instead used. The UPO panel enzymes 7 and 19 were left out since they appeared inactive with all studied substrates.

## Conflict of Interests

The authors declare no conflict of interest.

## Supporting information

As a service to our authors and readers, this journal provides supporting information supplied by the authors. Such materials are peer reviewed and may be re‐organized for online delivery, but are not copy‐edited or typeset. Technical support issues arising from supporting information (other than missing files) should be addressed to the authors.

Supporting Information

## Data Availability

The data that support the findings of this study are available from the corresponding author upon reasonable request.
