# Supplementary material for: Exploring Unspecific Peroxygenase Selectivity with Diverse Hydrocarbon Substrates
Source: ChemistryOpen. 2025 Jun 9;14(11):e202400521. doi: 10.1002/open.202400521 (PMC12598796; doi:10.1002/open.202400521)
Supplement: Supplementary file 1 — Supporting Information [file OPEN-14-e202400521-s001.pdf]

ChemistryOpen

Supporting Information

Exploring Unspecific Peroxygenase Selectivity with Diverse Hydrocarbon Substrates

Essi Rytkönen, Nina Hakulinen, Janne Jänis, and Juha Rouvinen*

Supporting information

Exploring Unspecific Peroxygenase Selectivity with Diverse Hydrocarbon Substrates

Essi Rytkönen, Nina Hakulinen, Janne Jänis and Juha Rouvinen*

Department of Chemistry, University of Eastern Finland, P.O. Box 111, FI-80101 Joensuu, Finland

*Corresponding author; email: juha.rouvinen@uef.fi

Contents:

Figure S1. Reaction protocol for UPO transformations

Table S1. Identified products for UPO panel with α-pinene

Table S2. Retention index data for α-pinene product identification

Table S3. Retention index data for (*S*)-limonene product identification

Table S4. Identified products for UPO panel with (*S*)-limonene

Figure S2. Activity profiles for panel UPOs

Figures S3-S8. Example chromatograms for the oxyfunctionalizations of all substrates and mass spectra for ethylbenzene and styrene biotransformations

Figure S9. Reaction routes for ethylbenzene oxyfunctionalization

Figure S10. Reaction route for styrene oxyfunctionalization

Figure S11. Reaction routes for toluene oxyfunctionalization

Figure S12. Reaction routes for cyclohexene oxyfunctionalization

Figure S13. Reaction routes for α-pinene oxyfunctionalization

Figure S14. Reaction routes for (S)-limonene oxyfunctionalization

References

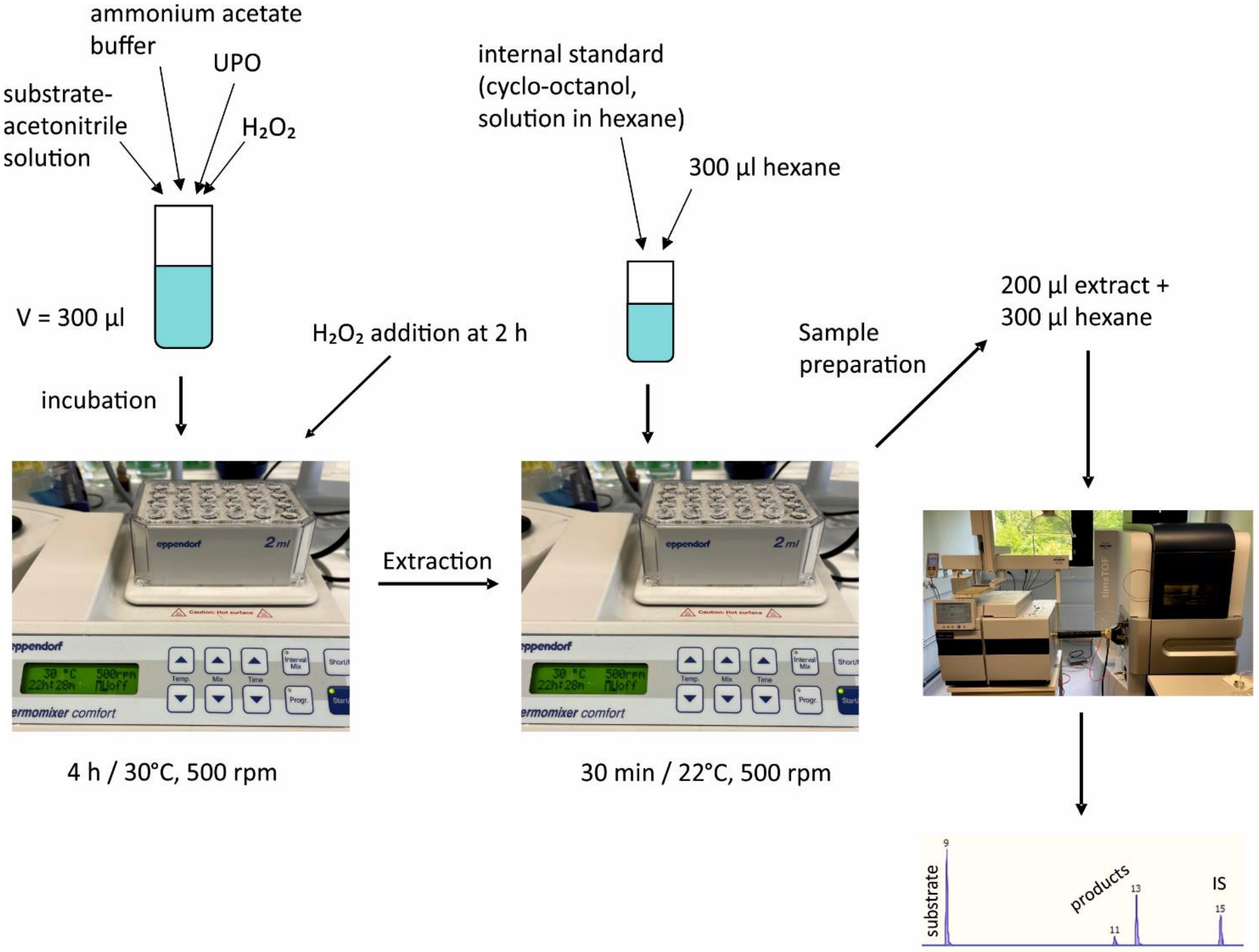

Figure S1. Reaction protocol for UPO transformations. More detailed information can be found in the Experimental section.

Table S1. Identified products and their relative amounts (%) for the UPO panel enzymes with α-pinene as the substrate.

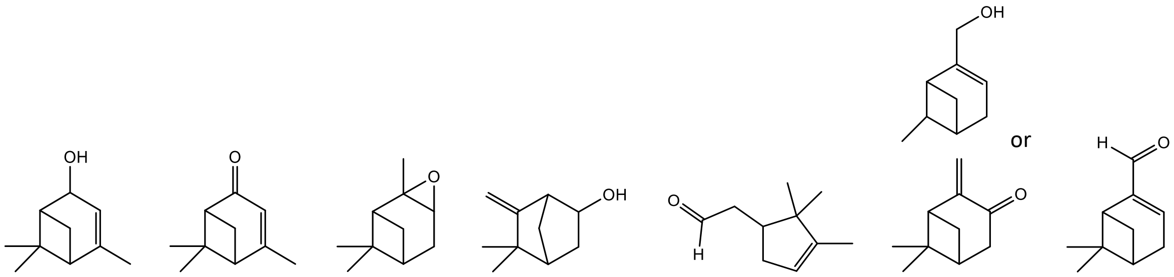

UPO	Verbenol*	Verbenone*	α-pinene oxide	Camphenol	Campholenic aldehyde	Product 6	Myrtenal
1	-	-	-	-	-	-	-
2[2]	-	0–23	0–1	0–4	21–26	50–75	-
3[2]	-	33	-	-	0–13	54–59	0–8
4	-	-	-	-	-	-	-
5[2]	-	22–23	0–1	-	19–22	44–50	8–10
6	-	-	-	-	-	-	-
8	-	-	-	-	-	-	-
9	-	-	-	-	-	-	-
10[2]	-	-	2–4	-	0–29	0–67	0–98
11[2]	7–100	0–23	-	-	0–21	0–36	0–13
12[2]	-	-	100	-	-	-	-
13[1]	58–66	0–24	-	-	18–34	-	-
14[2]	-	-	3–10	-	37–90	0–60	-
15[2]	-	-	-	-	39–65	0–61	0–35
16[2]	-	0–24	-	-	0–17	0–50	0–9
17	-	n.d.	-	n.d.	-	n.d.	-
18[1]	20–22	21–23	-	19	13–19	20–25	-
20[2]	-	0–48	0–3	-	35–100	-	0–14
21[2]	-	17–74	-	3–5	-	21–80	-
22	-	-	-	-	-	-	-
23[2]	-	26–33	3–5	-	0–12	53–57	0–11
24[1]	35–100	0–37	-	-	-	-	0–28
13M1[1]	50–61	0–27	-	-	0–39	-	0–23
13M2[1]	69–73	-	-	-	0–27	-	0–31
13M3[1]	57–73	0–26	-	-	18–27	-	-
13M4[1]	48–73	0–24	-	-	0–15	0–13	0–27
13M5[1]	50–59	0–21	-	-	15–28	0–14	0–14
13M6[1]	30–100	-	-	-	0–70	-	-

[1] = The product amounts (%) were calculated from FID chromatogram areas, [2] = the product amounts were calculated from EIC *m/z* 153.2 – 155.2 chromatogram areas, * = identified based on authentic standard.

Table S2. **Retention index data for the identification of α-pinene reaction products**. Published retention index data has been obtained from Pubchem for semi-standard non-polar column.[1]

Peak	LRI	Possible products (RI[1])	Identification	Notes
1	1099	α-pinene oxide (1097)	α-pinene oxide	
2	1118	Camphenol (1111)	Camphenol	
3	1127	Campholenic aldehyde (1124)	Campholenic aldehyde	MS similar to MS in NIST database[2]
4	1144	Verbenol (1144)	Verbenol	Identified by authentic standard
5	1173	Myrtenol (1182)<br>Pinocarvone (1161)	Unidentified	Presence of myrtenal would indicate myrtenol
6	1201	Myrtenal (1192)<br>α-terpineol (1204)	Myrtenal	α-terpineol was excluded based MS of standards
7	1211	Verbenone (1206)	Verbenone	Identified by authentic standard

Table S3. **Retention index data for the identification of (*S*)-limonene reaction products.** Published retention index data has been obtained from Pubchem for semi-standard non-polar column or from NIST database.[1,2]

Peak	LRI	Possible products (RI[1])	Identification	Notes
1	1136	1,2-epoxylimonene (1134)	1,2-epoxylimonene enantiomer	
2	1140	1,2-epoxylimonene (1134)	1,2-epoxylimonene enantiomer	MS identical to peak 1
3	1198	8,9-epoxylimonene (1199)	8,9-epoxylimonene enantiomer	
4	1201	8,9-epoxylimonene (1199)<br>Isopiperitenol (1210)	8,9-epoxylimonene enantiomer	MS identical to peak 3
5	1221	Carveol (1219)	Carveol	Identified by authentic standard
6	1279	Perillyl aldehyde (1276)<br>Isopiperitenone (1273)	Perillyl aldehyde	Isopiperitone was not identified in experiments
7	1296	Limonene dioxide (1294)<br>*p*-menth-1-en-9-ol (1298)	Limonene dioxide	MS more similar to dioxide MS in NIST database[2]
8	1301	Perillyl alcohol (1297)<br>*p*-menth-1-en-9-ol (1298)	Perillyl alcohol	
9	1322	Limonene 1,2-diol (1328)<br>Limonene aldehyde (1322[2])	Unidentified	MS in NIST database[2] did not correspond to either one

Table S4. Reaction products and their relative amounts (%) for panel UPOs with (*S*)-limonene as the substrate.

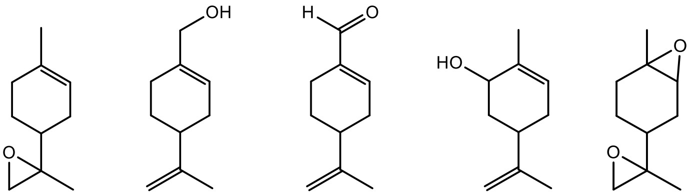

UPO	8,9-epoxy-limonene	Perillyl alcohol	Perillyl aldehyde	Carveol*	Limonene dioxide
1	-	-	-	-	-
2[2]	0–88	12–24	-	0–76	-
3	-	-	-	-	-
4	-	-	-	-	-
5[2]	0–61	39–100	-	-	-
6[2]	100	-	-	-	-
8[2]	35–38	0–3	-	62–63	-
9[2]	29–100	-	-	0–71	-
10[2]	0–41	8–100	-	0–51	-
11[2]	41–93	2–7	-	0–58	-
12	-	-	-	-	-
13[2]	0–73	27–100	-	-	-
14[2]	0–72	28–100	-	-	-
15[2]	38–96	3–4	-	0–58	-
16[2]	65–67	0–2	-	30–35	-
17[2]	0–81	19–100	-	-	-
18[1]	76–85	5–8	8–19	-	-
20[2]	88–94	6–12	-	-	-
21[1]	0–53	23–100	18	-	0–6
22[2]	0–29	-	-	71–100	-
23[2]	-	70–100	30	-	-
24[2]	0–78	22–100	-	-	-
13M1[2]	0–74	26–100	-	-	-
13M2[2]	0–36	11–100	-	0–53	-
13M3[2]	0–59	41–100	-	-	-
13M4[2]	0–62	38–100	-	-	-
13M5[2]	-	100	-	-	-
13M6[2]	-	100	-	-	-

[1] = The product amounts (%) were calculated from FID chromatogram areas, [2] = the product amounts were calculated from EIC *m/z* 153.2 – 155.2 and *m/z* 135 chromatogram areas, * = identified based on authentic standard.

UPO1	Hydrogen abstraction	Epoxi-dation	Aromatic	Aliphatic	Allylic	Over-oxidation
ethylbenzene	**	-	-	**	-	*
styrene	-	**	-	**	-	-
toluene	-	**	**	-	-	-
octane	***	-	-	***	-	-
cyclohexene	-	****	-	****	-	-
limonene	-	-	-	-	-	-
pinene	-	-	-	-	-	-

UPO2	Hydrogen abstraction	Epoxi-dation	Aromatic	Aliphatic	Allylic	Over-oxidation
ethylbenzene	**	**	**	**	-	**
styrene	-	***	-	***	-	-
toluene	-	***	***	-	-	*
octane	**	-	-	**	-	**
cyclohexene	-	****	-	****	-	-
limonene	**	*	-	***	**	-
pinene	**	***	-	*****	**	*

UPO3	Hydrogen abstraction	Epoxi-dation	Aromatic	Aliphatic	Allylic	Over-oxidation
ethylbenzene	**	**	**	**	-	**
styrene	-	***	-	***	-	-
toluene	-	***	***	-	-	*
octane	***	-	-	***	-	*
cyclohexene	-	****	-	****	-	-
limonene	-	-	-	-	-	-
pinene	***	*	-	****	***	**

UPO4	Hydrogen abstraction	Epoxi-dation	Aromatic	Aliphatic	Allylic	Over-oxidation
ethylbenzene	**	-	-	**	-	*
styrene	-	*	-	*	-	-
toluene	-	**	**	-	-	-
octane	***	-	-	***	-	-
cyclohexene	-	****	-	****	-	-
limonene	-	-	-	-	-	-
pinene	-	-	-	-	-	-

UPO5	Hydrogen abstraction	Epoxi-dation	Aromatic	Aliphatic	Allylic	Over-oxidation
ethylbenzene	**	**	**	**	-	**
styrene	-	****	-	****	-	-
toluene	-	***	***	-	-	*
octane	**	-	-	**	-	**
cyclohexene	*	****	-	****	*	*
limonene	*	*	-	**	*	-
pinene	***	**	-	*****	***	**

UPO6	Hydrogen abstraction	Epoxi-dation	Aromatic	Aliphatic	Allylic	Over-oxidation
ethylbenzene	**	-	-	**	-	*
styrene	-	**	-	**	-	-
toluene	-	**	**	-	-	-
octane	*	-	-	*	-	-
cyclohexene	*	****	-	****	*	-
limonene	-	*	-	*	-	-
pinene	-	-	-	-	-	-

UPO7	Hydrogen abstraction	Epoxi-dation	Aromatic	Aliphatic	Allylic	Over-oxidation
ethylbenzene	-	-	-	-	-	-
styrene	-	-	-	-	-	-
toluene	-	-	-	-	-	-
octane	-	-	-	-	-	-
cyclohexene	-	-	-	-	-	-
limonene	-	-	-	-	-	-
pinene	-	-	-	-	-	-

UPO8	Hydrogen abstraction	Epoxi-dation	Aromatic	Aliphatic	Allylic	Over-oxidation
ethylbenzene	**	*	*	**	-	**
styrene	-	*	-	*	-	-
toluene	-	**	**	-	-	*
octane	**	-	-	**	-	**
cyclohexene	-	****	-	****	-	-
limonene	**	*	-	***	**	-
pinene	-	-	-	-	-	-

UPO9	Hydrogen abstraction	Epoxi-dation	Aromatic	Aliphatic	Allylic	Over-oxidation
ethylbenzene	**	*	*	**	-	**
styrene	-	***	-	***	-	-
toluene	*	***	***	*	-	*
octane	**	-	-	**	-	*
cyclohexene	*	****	-	****	*	-
limonene	*	*	-	**	*	-
pinene	-	-	-	-	-	-

Figure S2. Activity profiles for individual UPOs in the panel for all substrates. Number of asterisks in the case of ethylbenzene, toluene, limonene and pinene signifies the number of products of certain type. Allylic products are included in aliphatic products for terpenes. For octane, denotations mean *= oxidation to one carbon atom, **=oxidation to two carbons, ***= oxidation to three carbons, and in overoxidation column *= one carbon and **= 2 or 3 carbons (as 2-octanone and 4-octanol could not be distinguished). For styrene, average of total product FID areas was calculated and relative product amounts of UPOs compared to highest product amount (UPO14) were calculated, and denoted *= 0–25%, **= 25–50%, ***=50–75% and ****= 75–100%. Similarly, relative amounts of cyclohexene products were calculated from FID chromatogram of single UPO and denoted *= 0–25%, **= 25–50%, ***=50–75% and ****= 75–100% of total products. Product types that were observed only in EIC *m/z* 97 chromatograms in minor amounts for cyclohexene are colored in orange.

UPO10	Hydrogen abstraction	Epoxi-dation	Aromatic	Aliphatic	Allylic	Over-oxidation
ethylbenzene	**	**	**	**	-	**
styrene	-	****	-	****	-	-
toluene	-	***	***	-	-	*
octane	**	-	-	**	-	**
cyclohexene	*	****	-	****	*	*
limonene	**	*	-	***	**	-
pinene	**	**	-	****	**	*

UPO11	Hydrogen abstraction	Epoxi-dation	Aromatic	Aliphatic	Allylic	Over-oxidation
ethylbenzene	**	**	**	**	-	**
styrene	-	**	-	**	-	-
toluene	-	**	**	-	-	*
octane	***	-	-	***	-	**
cyclohexene	-	****	-	****	-	-
limonene	**	*	-	***	**	-
pinene	****	*	-	*****	****	**

UPO12	Hydrogen abstraction	Epoxi-dation	Aromatic	Aliphatic	Allylic	Over-oxidation
ethylbenzene	**	-	-	**	-	*
styrene	-	***	-	***	-	-
toluene	*	***	***	*	-	**
octane	**	-	-	**	-	-
cyclohexene	**	***	-	***	**	-
limonene	-	-	-	-	-	-
pinene	-	*	-	*	-	-

UPO13	Hydrogen abstraction	Epoxi-dation	Aromatic	Aliphatic	Allylic	Over-oxidation
ethylbenzene	**	**	**	**	-	**
styrene	-	***	-	***	-	-
toluene	**	***	***	**	-	**
octane	***	-	-	***	-	**
cyclohexene	*	****	-	****	*	*
limonene	*	*	-	**	*	-
pinene	**	*	-	***	**	*

UPO14	Hydrogen abstraction	Epoxi-dation	Aromatic	Aliphatic	Allylic	Over-oxidation
ethylbenzene	**	**	**	**	-	**
styrene	-	****	-	****	-	-
toluene	***	*	*	***	-	**
octane	**	-	-	**	-	**
cyclohexene	*	****	-	****	*	*
limonene	*	*	-	**	*	-
pinene	*	**	-	***	*	-

UPO15	Hydrogen abstraction	Epoxi-dation	Aromatic	Aliphatic	Allylic	Over-oxidation
ethylbenzene	**	**	**	**	-	**
styrene	-	**	-	**	-	-
toluene	-	***	***	-	-	*
octane	***	-	-	***	-	**
cyclohexene	*	****	-	****	*	-
limonene	**	*	-	***	**	-
pinene	**	*	-	***	**	*

UPO16	Hydrogen abstraction	Epoxi-dation	Aromatic	Aliphatic	Allylic	Over-oxidation
ethylbenzene	**		-	**	-	*
styrene	-	***	-	***	-	-
toluene	-	-	-	-	-	-
octane	-	-	-	-	-	-
cyclohexene	*	****	-	****	*	*
limonene	**	*	-	***	**	-
pinene	***	*	-	****	***	**

UPO17	Hydrogen abstraction	Epoxi-dation	Aromatic	Aliphatic	Allylic	Over-oxidation
ethylbenzene	**	**	**	**	-	**
styrene	-	***	-	***	-	-
toluene	*	***	***	*	-	**
octane	**	-	-	**	-	**
cyclohexene	*	****	-	****	*	*
limonene	*	*	-	**	*	-
pinene	**	*	-	***	**	*

UPO18	Hydrogen abstraction	Epoxi-dation	Aromatic	Aliphatic	Allylic	Over-oxidation
ethylbenzene	***	-	-	***	-	*
styrene	-	****	-	****	-	-
toluene	**	-	-	**	-	*
octane	**	-	-	**	-	*
cyclohexene	**	***	-	***	**	**
limonene	**	*	-	***	**	*
pinene	***	**	-	*****	***	*

Figure S2. Activity profiles for individual UPOs in the panel for all substrates. *Continued.*

UPO19	Hydrogen abstraction	Epoxi-dation	Aromatic	Aliphatic	Allylic	Over-oxidation
ethylbenzene	-	-	-	-	-	-
styrene	-	-	-	-	-	-
toluene	-	-	-	-	-	-
octane	-	-	-	-	-	-
cyclohexene	-	-	-	-	-	-
limonene	-	-	-	-	-	-
pinene	-	-	-	-	-	-

UPO20	Hydrogen abstraction	Epoxi-dation	Aromatic	Aliphatic	Allylic	Over-oxidation
ethylbenzene	**	**	**	**	-	**
styrene	-	***	-	***	-	-
toluene	-	***	***	-	-	*
octane	**	-	-	**	-	**
cyclohexene	*	****	-	****	*	-
limonene	*	*	-	**	*	-
pinene	**	**	-	****	**	**

UPO21	Hydrogen abstraction	Epoxi-dation	Aromatic	Aliphatic	Allylic	Over-oxidation
ethylbenzene	****	-	-	****	-	**
styrene	-	****	-	****	-	-
toluene	**	-	-	**	-	*
octane	-	-	-	-	-	-
cyclohexene	**	***	-	***	**	*
limonene	**	**	-	****	**	**
pinene	**	*	-	***	**	*

UPO22	Hydrogen abstraction	Epoxi-dation	Aromatic	Aliphatic	Allylic	Over-oxidation
ethylbenzene	**	**	**	**	-	**
styrene	-	**	-	**	-	-
toluene	-	-	-	-	-	-
octane	***	-	-	***	-	**
cyclohexene	-	****	-	****	-	-
limonene	*	*	-	**	*	-
pinene	-	-	-	-	-	-

UPO23	Hydrogen abstraction	Epoxi-dation	Aromatic	Aliphatic	Allylic	Over-oxidation
ethylbenzene	**	*	*	**	-	**
styrene	-	***	-	***	-	-
toluene	**	***	**	***	-	**
octane	**	-	-	**	-	*
cyclohexene	-	****	-	****	-	-
limonene	**	-	-	**	**	*
pinene	***	**	-	*****	***	**

UPO24	Hydrogen abstraction	Epoxi-dation	Aromatic	Aliphatic	Allylic	Over-oxidation
ethylbenzene	**	**	**	**	-	**
styrene	-	****	-	****	-	-
toluene	**	***	***	**	-	**
octane	**	-	-	**	-	**
cyclohexene	*	****	-	****	*	*
limonene	*	*	-	**	*	-
pinene	***	-	-	***	***	**

UPO13M1	Hydrogen abstraction	Epoxi-dation	Aromatic	Aliphatic	Allylic	Over-oxidation
ethylbenzene	**	**	**	**	-	**
styrene	-	***	-	***	-	-
toluene	*	***	***	*	-	**
octane	**	-	-	**	-	**
cyclohexene	*	****	-	****	*	*
limonene	*	*	-	**	*	-
pinene	***	*	-	****	***	**

UPO13M2	Hydrogen abstraction	Epoxi-dation	Aromatic	Aliphatic	Allylic	Over-oxidation
ethylbenzene	**	**	**	**	-	**
styrene	-	***	-	***	-	-
toluene	*	***	***	*	-	**
octane	**	-	-	**	-	**
cyclohexene	*	****	-	****	*	*
limonene	**	*	-	***	**	-
pinene	**	*	-	***	**	*

UPO13M3	Hydrogen abstraction	Epoxi-dation	Aromatic	Aliphatic	Allylic	Over-oxidation
ethylbenzene	**	**	**	**	-	**
styrene	-	****	-	****	-	-
toluene	*	***	***	*	-	**
octane	**	-	-	**	-	**
cyclohexene	*	****	-	****	*	*
limonene	*	*	-	**	*	-
pinene	**	*	-	***	**	*

UPO13M4	Hydrogen abstraction	Epoxi-dation	Aromatic	Aliphatic	Allylic	Over-oxidation
ethylbenzene	**	**	**	**	-	**
styrene	-	****	-	****	-	-
toluene	*	***	***	*	-	**
octane	**	-	-	**	-	**
cyclohexene	*	****	-	****	*	*
limonene	*	*	-	**	*	-
pinene	****	*	-	*****	****	**

UPO13M5	Hydrogen abstraction	Epoxi-dation	Aromatic	Aliphatic	Allylic	Over-oxidation
ethylbenzene	**	**	**	**	-	**
styrene	-	***	-	***	-	-
toluene	*	***	***	*	-	**
octane	**	-	-	**	-	**
cyclohexene	*	****	-	****	*	*
limonene	*	-	-	*	*	-
pinene	****	*	-	*****	****	**

UPO13M6	Hydrogen abstraction	Epoxi-dation	Aromatic	Aliphatic	Allylic	Over-oxidation
ethylbenzene	**	**	**	**	-	**
styrene	-	****	-	****	-	-
toluene	*	***	***	*	-	**
octane	**	-	-	**	-	**
cyclohexene	*	****	-	****	*	*
limonene	*	-	-	*	*	-
pinene	*	*	-	**	*	-

Figure S2. Activity profiles for individual UPOs in the panel for all substrates. *Continued.*

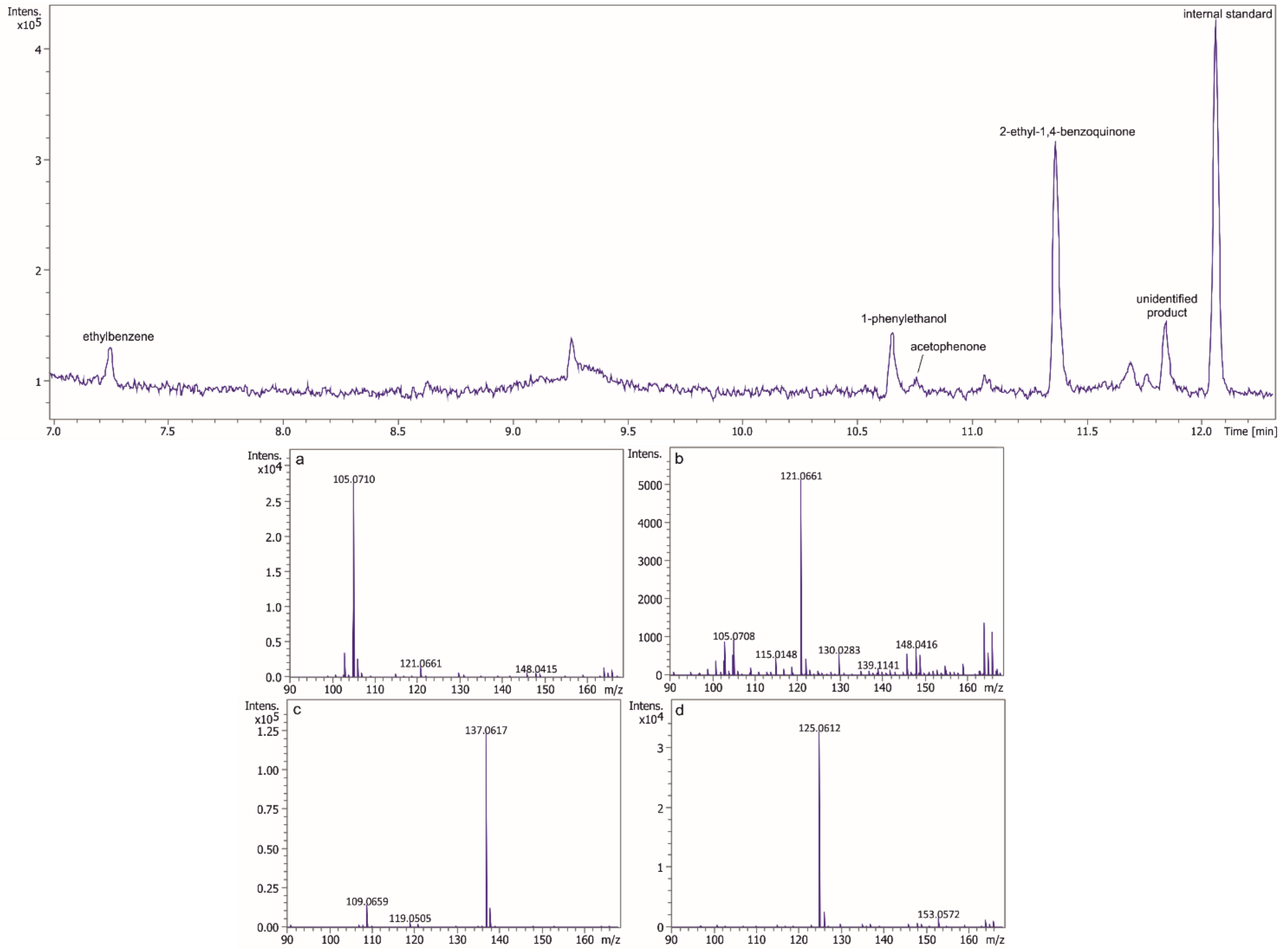

Figure S3. Example TIC chromatogram and mass spectra for ethylbenzene biotransformation with UPO13. The mass spectra are for a) 1-phenylethanol, b) acetophenone, c) 2-ethyl-1,4-benzoquinone and d) the unidentified product.

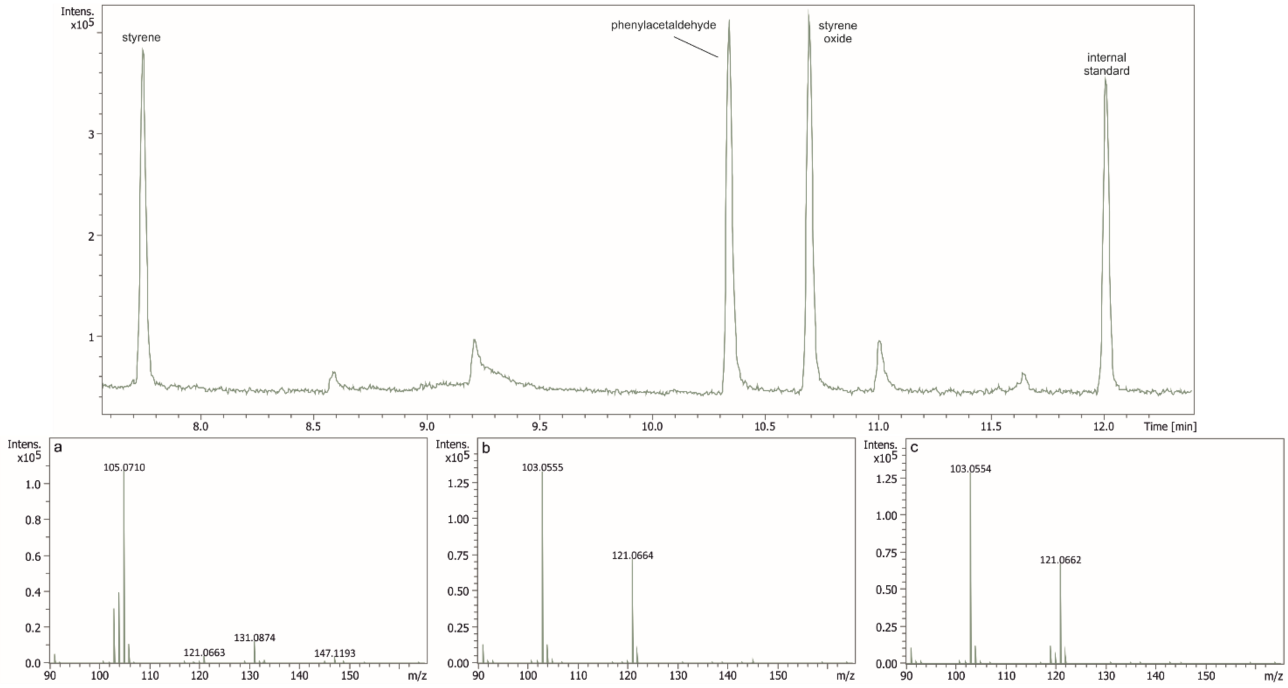

Figure S4. Example TIC chromatogram and mass spectra for styrene oxyfunctionalization by UPO13. The mass spectra are for a) styrene, b) phenylacetaldehyde and c) styrene oxide.

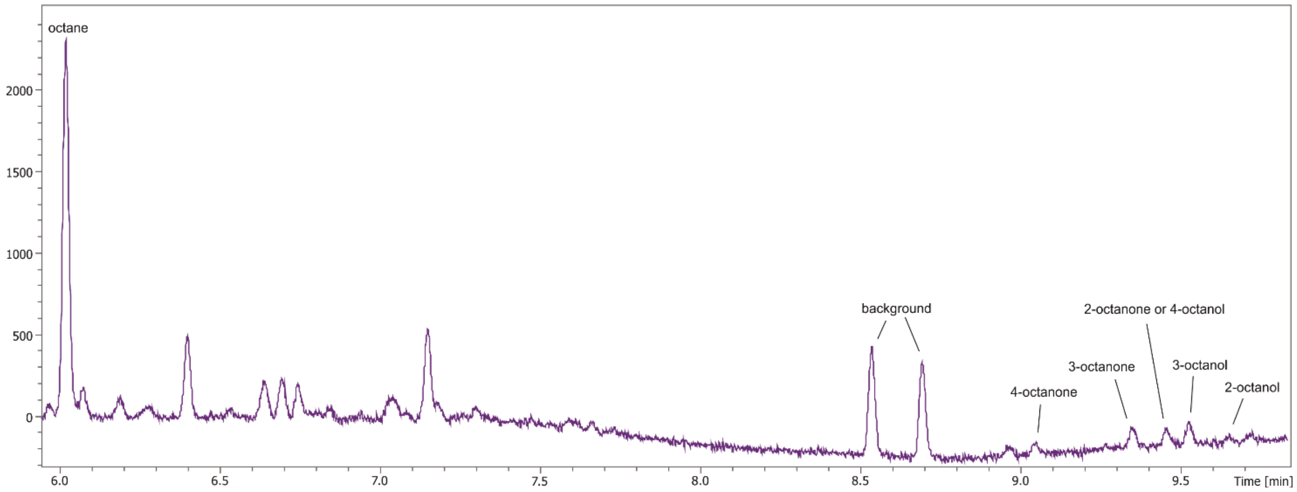

Figure S5. Example FID chromatogram for octane biotransformation by UPO11.

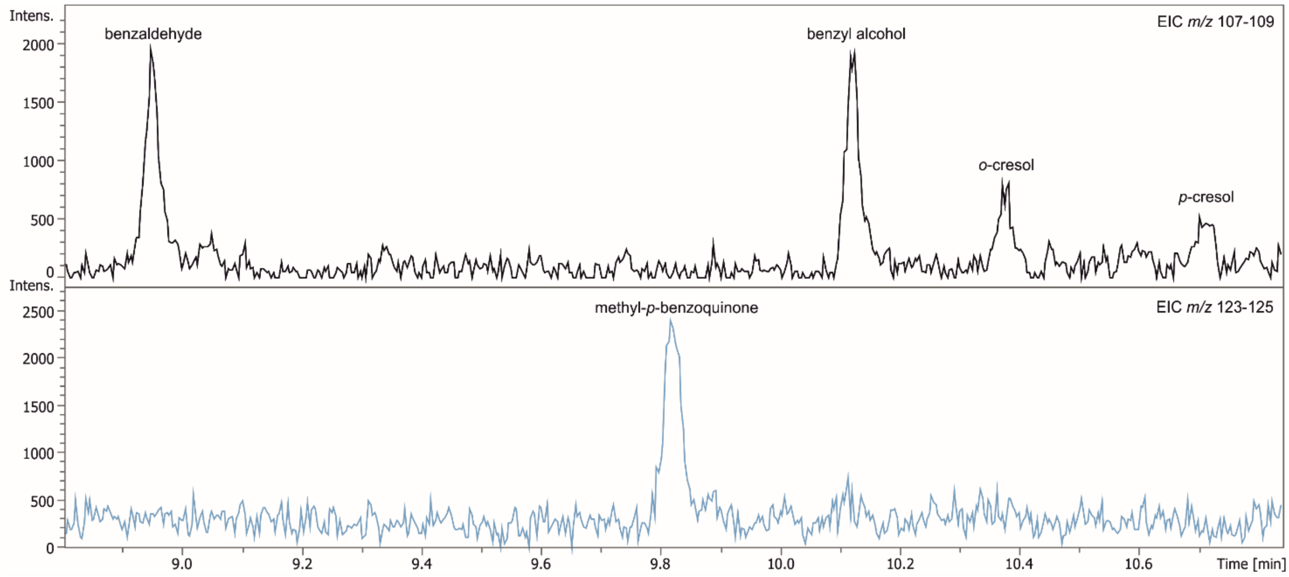

Figure S6. Example EIC chromatograms for toluene oxyfunctionalization by UPO14. The black chromatogram corresponds to EIC 107-109 and blue one to EIC 123-125.

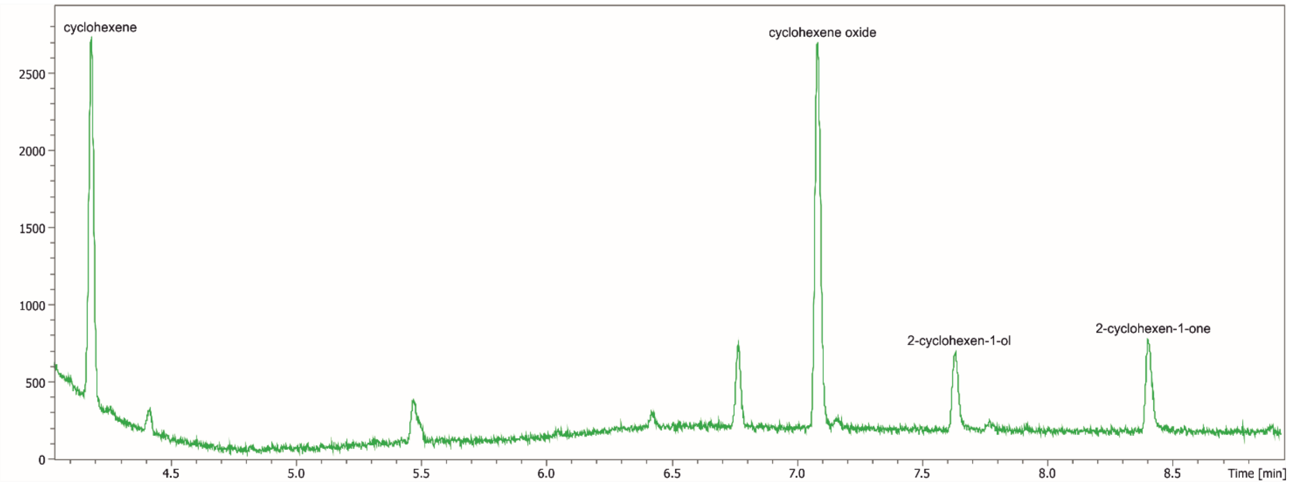

Figure S7. Example FID chromatogram of cyclohexene biotransformation with UPO18.

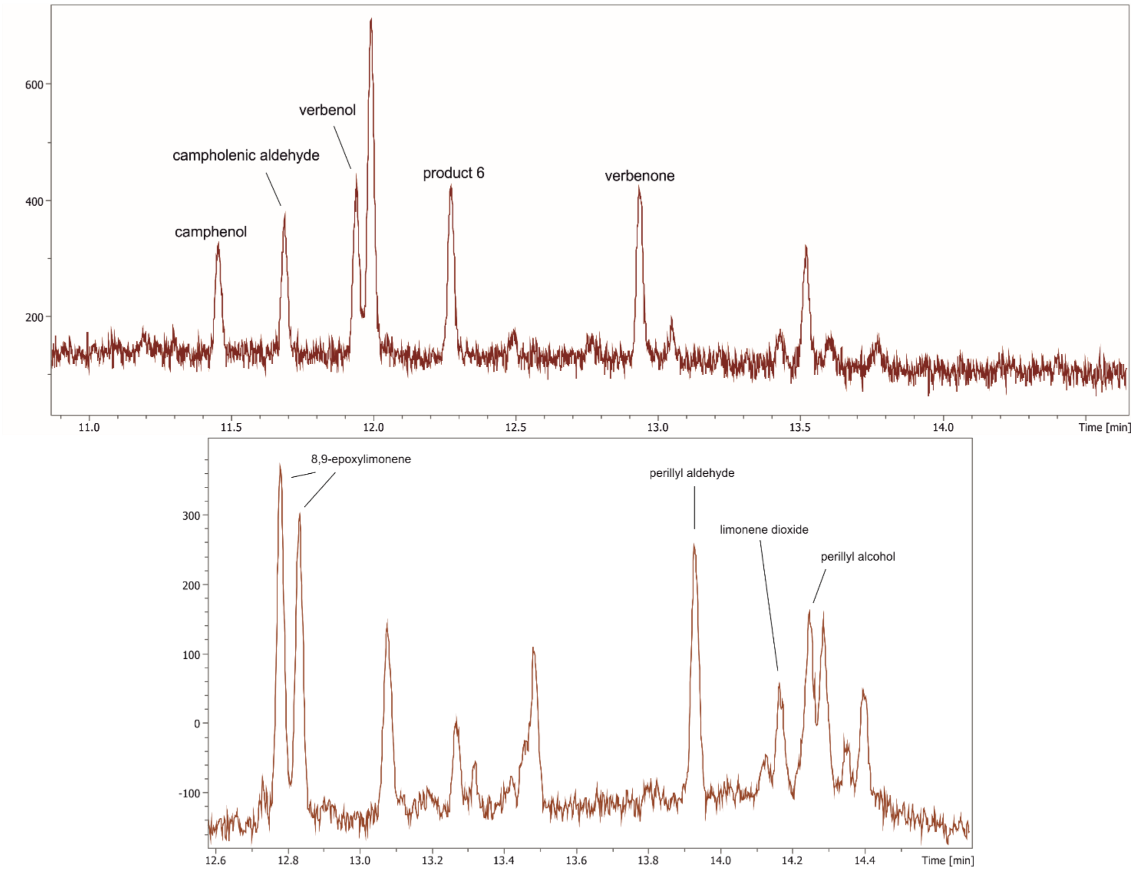

Figure S8. Example FID chromatograms for the terpene biotransformations. The chromatogram on the top is for α-pinene oxyfunctionalization by UPO18 and the bottom chromatogram represents (*S*)-limonene biotransformation by UPO21.

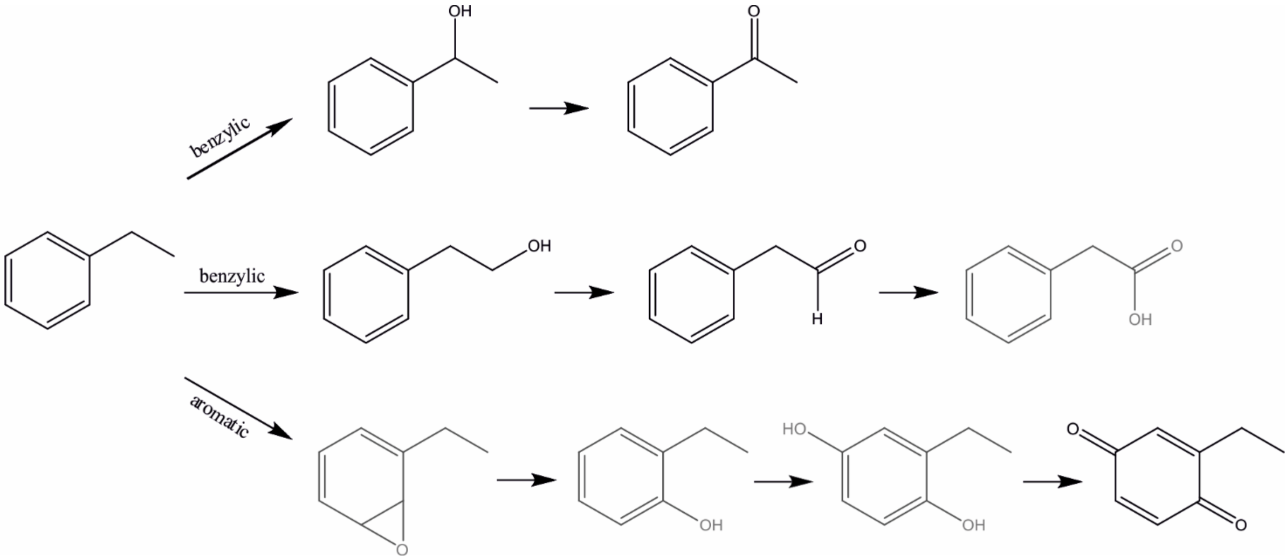

Figure S9. Proposed reaction routes for ethylbenzene oxidation products. Intermediates that were not observed in the reaction extracts are marked in gray.

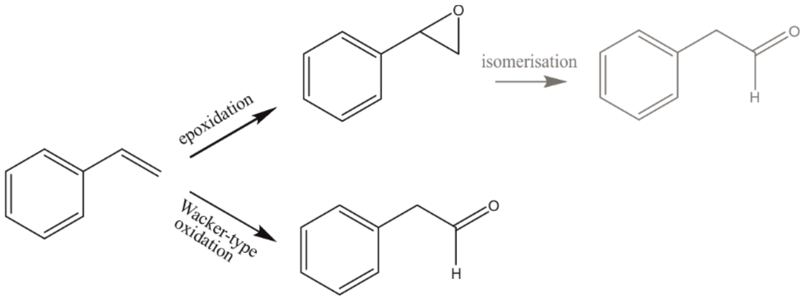

Figure S10. Formation of styrene oxidation products with UPOs. The other possible route to phenylacetaldehyde has been marked in gray.

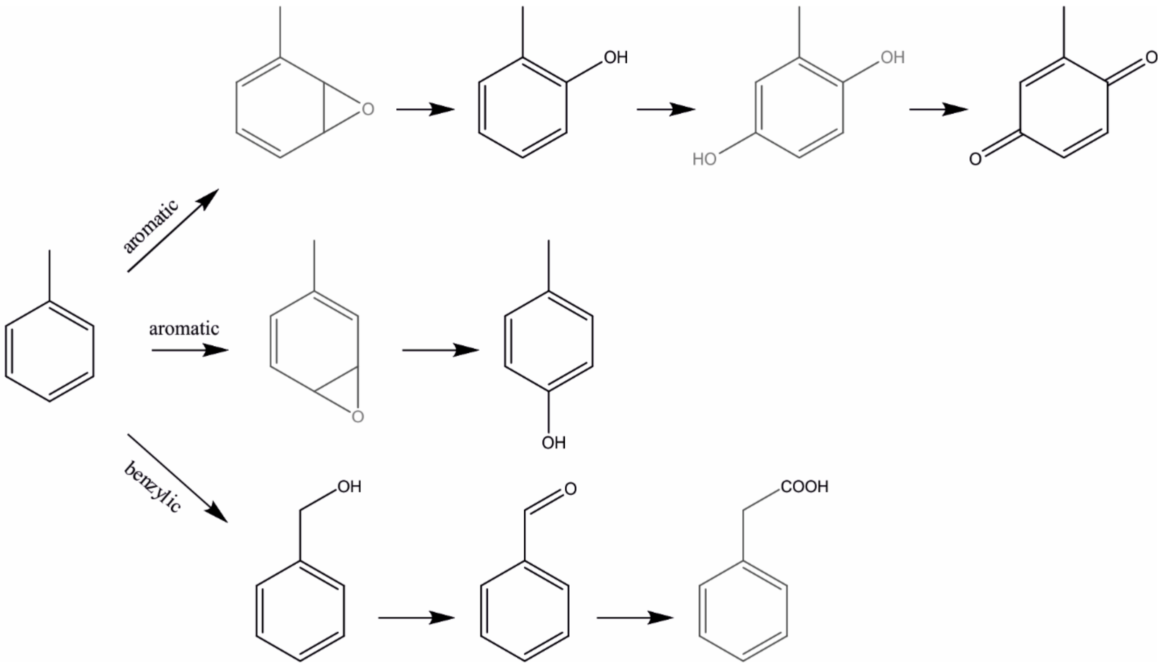

Figure S11. Proposed reaction routes for toluene oxyfunctionalization by UPOs. Intermediates and overoxidation product that were not observed are colored in gray.

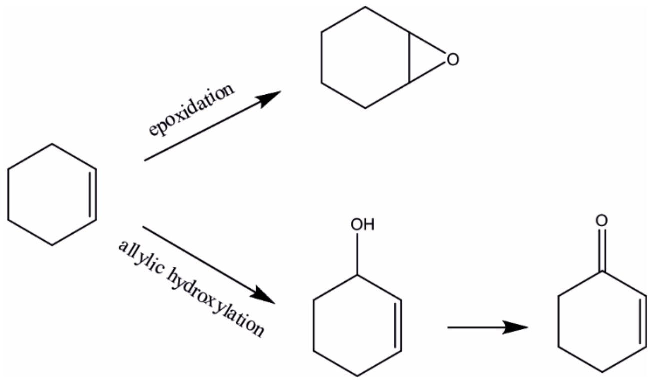

Figure S12. Reaction routes for cyclohexene oxyfunctionalization that were observed with the UPO panel.

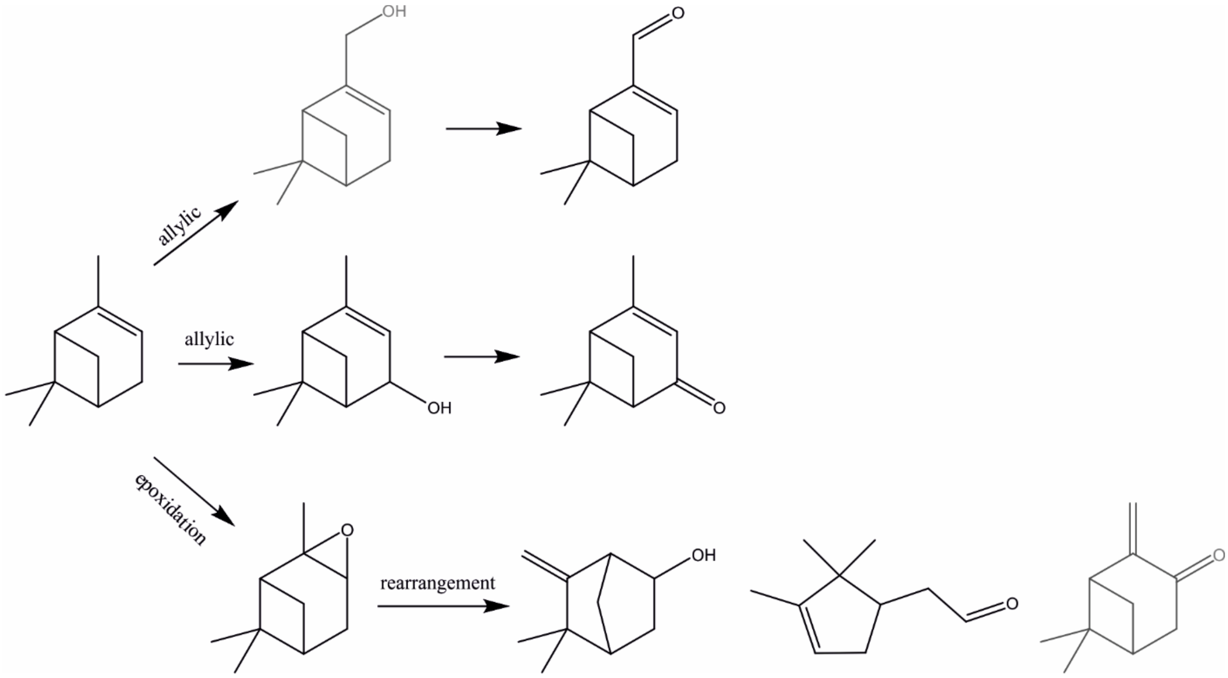

Figure S13. Reaction routes for proposed products in α-pinene oxyfunctionalization. Only products that were identified in the experiments are included in the figure. Two possible compounds based on retention indices for product 6 have been marked in gray.

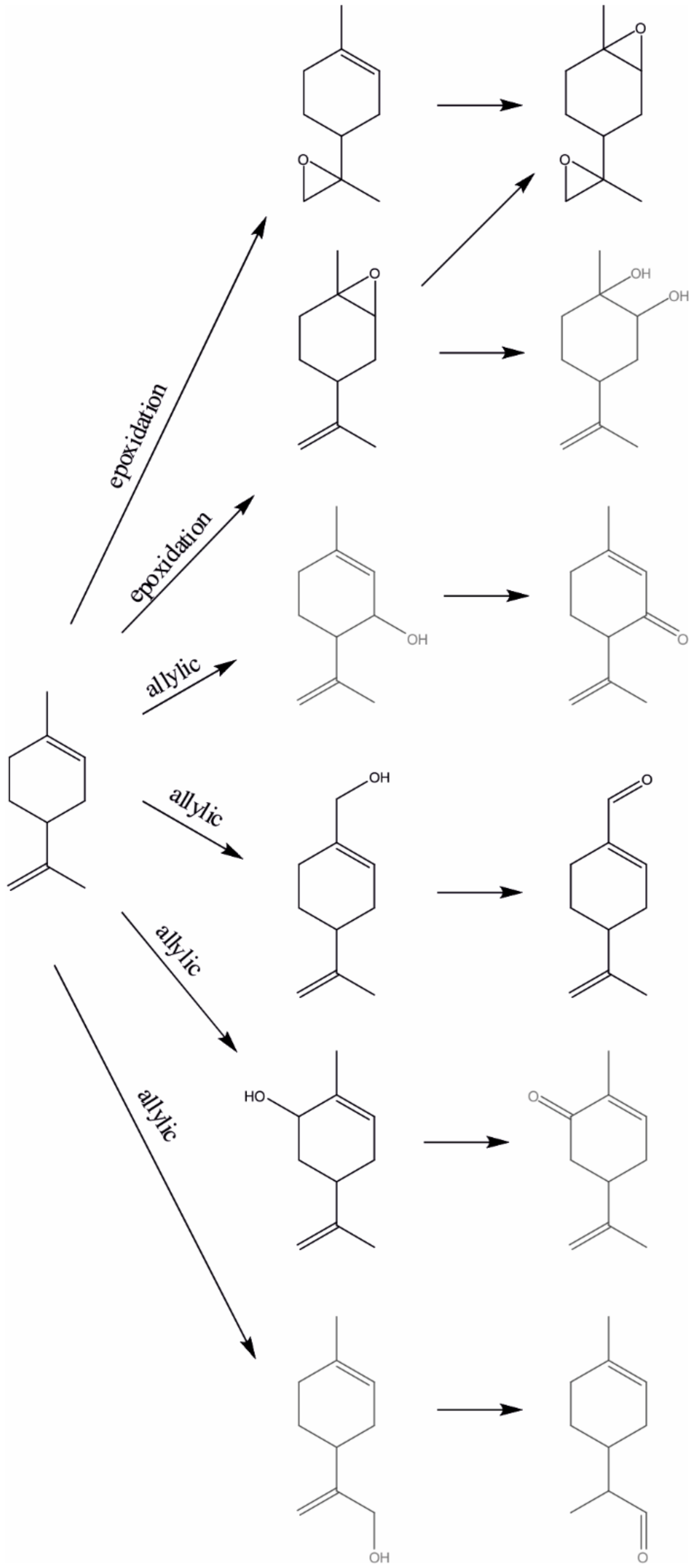

Figure S14. Reaction routes for possible products in (S)-limonene oxyfunctionalization. Products that were not detected or only tentatively identified are marked in gray.

References

(1) Kim, S.; Chen, J.; Cheng, T.; Gindulyte, A.; He, J.; He, S.; Li, Q.; Shoemaker, B. A.; Thiessen, P. A.; Yu, B.; Zaslavsky, L.; Zhang, J.; Bolton, E. E. PubChem 2023 Update. *Nucleic Acids Res.* **2023**, *51* (D1), D1373–D1380. https://doi.org/10.1093/NAR/GKAC956.

(2) *NIST Chemistry WebBook, NIST Standard Reference Database Number 69*; Linstrom, P. J., Mallard, W. G., Eds.; National Institute of Standards and Technology, Gaithersburg MD. https://doi.org/https://doi.org/10.18434/T4D303.
